# Virulence Factors of *Pseudomonas aeruginosa* Induce Both the Unfolded Protein and Integrated Stress Responses in Airway Epithelial Cells

**DOI:** 10.1371/journal.ppat.1004946

**Published:** 2015-06-17

**Authors:** Emily F. A. van ‘t Wout, Annemarie van Schadewijk, Ria van Boxtel, Lucy E. Dalton, Hanna J. Clarke, Jan Tommassen, Stefan J. Marciniak, Pieter S. Hiemstra

**Affiliations:** 1 Department of Pulmonology, Leiden University Medical Centre, Leiden, the Netherlands; 2 Cambridge Institute for Medical Research (CIMR), University of Cambridge, Cambridge, United Kingdom; 3 Department of Molecular Microbiology, Utrecht University, Utrecht, the Netherlands; University of Washington, UNITED STATES

## Abstract

*Pseudomonas aeruginosa* infection can be disastrous in chronic lung diseases such as cystic fibrosis and chronic obstructive pulmonary disease. Its toxic effects are largely mediated by secreted virulence factors including pyocyanin, elastase and alkaline protease (AprA). Efficient functioning of the endoplasmic reticulum (ER) is crucial for cell survival and appropriate immune responses, while an excess of unfolded proteins within the ER leads to “ER stress” and activation of the “unfolded protein response” (UPR). Bacterial infection and Toll-like receptor activation trigger the UPR most likely due to the increased demand for protein folding of inflammatory mediators. In this study, we show that cell-free conditioned medium of the PAO1 strain of *P*. *aeruginosa*, containing secreted virulence factors, induces ER stress in primary bronchial epithelial cells as evidenced by splicing of *XBP1* mRNA and induction of *CHOP*, *GRP78* and *GADD34* expression. Most aspects of the ER stress response were dependent on TAK1 and p38 MAPK, except for the induction of *GADD34* mRNA. Using various mutant strains and purified virulence factors, we identified pyocyanin and AprA as inducers of ER stress. However, the induction of *GADD34* was mediated by an ER stress-independent integrated stress response (ISR) which was at least partly dependent on the iron-sensing eIF2α kinase HRI. Our data strongly suggest that this increased *GADD34* expression served to protect against *Pseudomonas*-induced, iron-sensitive cell cytotoxicity. In summary, virulence factors from *P*. *aeruginosa* induce ER stress in airway epithelial cells and also trigger the ISR to improve cell survival of the host.

## Author Summary

*Pseudomonas aeruginosa* causes a devastating infection when it affects patients with cystic fibrosis or other chronic lung diseases. It often causes chronic infection due to its resistance to antibiotic treatment and its ability to form biofilms in these patients. The toxic effects of *P*. *aeruginosa* are largely mediated by secreted virulence factors. Efficient functioning of the endoplasmic reticulum is crucial for cell survival and appropriate immune responses, while its dysfunction causes stress and activation of the unfolded protein response. In this study, we found that virulence factors secreted by *P*. *aeruginosa* trigger the unfolded protein response in human cells by causing endoplasmic reticulum stress. In addition, secreted virulence factors activate the integrated stress response via a parallel independent pathway. Both stress pathways lead to the induction of the protein GADD34, which appears to provide protection against the toxic effects of the secreted virulence factors.

## Introduction

The Gram-negative bacterium *Pseudomonas aeruginosa* is an opportunistic pathogen that increases morbidity and mortality in chronic lung diseases, such as cystic fibrosis (CF) and chronic obstructive pulmonary disease (COPD; GOLD stages III-IV)) [1–3]. *P*. *aeruginosa* often causes chronic infection due to its ease of developing antibiotic resistance and its ability to form biofilms in these patients. Furthermore, its survival in the host in the early stages of infection is supported by the secretion of toxins and virulence factors, including pyocyanin and its proteases elastase and alkaline protease (AprA) (reviewed in [4, 5]). Interestingly, their production appears to be lower in the later stages of infection [6, 7]. Therefore, the specific role of these virulence factors in chronic infections is incompletely understood. Pyocyanin is a redox-active toxin that causes cellular senescence [8], ciliary dyskinesia [9], increased expression of IL-8 [10] and disruption of calcium homeostasis [11] in human lung epithelial cells. Pyocyanin inactivates α_1_-antitrypsin, thereby contributing to the protease-antiprotease imbalance found in CF lungs [12], while *P*. *aeruginosa* elastase additionally cleaves many proteins of the extra-cellular matrix, including collagen, fibrinogen and elastin, and opsonin receptors, thus contributing to the invasion of bacteria into the lung parenchyma [13]. AprA is thought to modulate the host response and prevent bacterial clearance by degrading proteins of the host immune system, including TNFα and complement factors [14–16].

*P*. *aeruginosa* requires iron both for its respiration and for biofilm formation [17, 18]. Competition with the host is fierce and so *P*. *aeruginosa* has evolved specific strategies to obtain iron [19]. It produces redox-active phenazine compounds to turn insoluble Fe^3+^ to the more soluble Fe^2+^, siderophores to scavenge iron and receptors for the uptake of iron-siderophore complexes, proteases to degrade host iron-binding proteins, and bacteriocins to eliminate competitors (reviewed in [19]). Moreover, iron availability regulates the production of virulence factors such as pyocyanin, AprA and exotoxin A [20].

**Fig 1:**
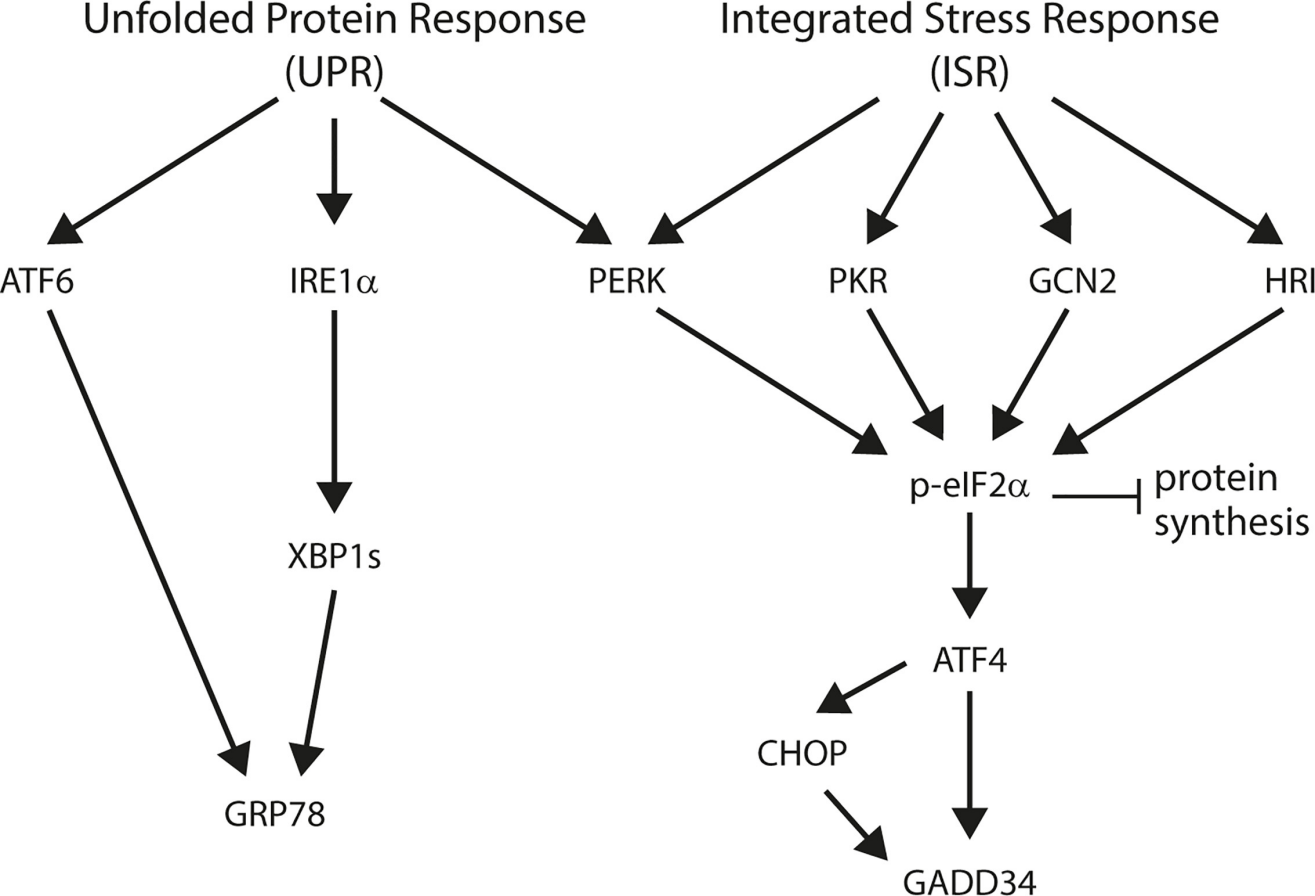
Schematic overview of the unfolded protein response (UPR) and integrated stress response (ISR).

The endoplasmic reticulum (ER) functions to fold secretory and membrane proteins and its quality control systems ensure that only properly folded proteins exit the organelle. Accumulation of incompletely folded proteins can impair ER homeostasis and induces “ER stress”, which activates intracellular signal transduction pathways collectively called the “unfolded protein response” (UPR; Fig 1). This response restores ER homeostasis by reducing the influx of new proteins into the lumen of the ER and by enhancing the organelle’s capacity to fold proteins; however, if the stress cannot be resolved then apoptotic cell death pathways are invoked (reviewed in [21]).

Three distinct sensors detect ER stress: protein kinase RNA (PKR)-like ER kinase (PERK), inositol-requiring enzyme 1 (IRE1) and activating transcription factor 6 (ATF6) [21]. Early during ER stress, the kinase PERK phosphorylates eukaryotic translation initiation factor 2 on its alpha subunit (eIF2α) causing the inhibition of protein synthesis and thus preventing the load on the ER from increasing further [22–24]. In addition, this promotes the translation of specific mRNAs, for example that encoding the transcription factor ATF4 [25]. One important target of ATF4 is the transcription factor called C/EBP homologous protein (CHOP), and both individually can trans-activate the *GADD34* gene [26]. GADD34 is a phosphatase that selectively dephosphorylates eIF2α, completing a negative feedback loop and enabling the translation of other targets of the UPR [27]. In parallel, IRE1 initiates the unconventional splicing of the mRNA encoding X-box binding protein-1 (XBP-1) [28]. Spliced *XBP-1* mRNA encodes an active transcription factor that, in concert with ATF6, induces expression of UPR genes, such as the chaperones GRP78 (also known as BiP) and GRP94 [28–30].

The phosphorylation of eIF2α is a point at which the responses to several forms of stress are integrated [31]. During ER stress, PERK phosphorylates eIF2α, but eIF2a can also be phosphorylated by PKR responding to double-stranded RNA during viral infection [32, 33], by GCN2 during amino acid starvation [25, 34, 35], and by HRI during iron deficiency (reviewed in [31]). For this reason, the events initiated by eIF2α phosphorylation have been termed the “integrated stress response” (ISR; Fig 1 and [36]).

Abnormal function of the ER has been implicated in the pathogenesis of many diseases, including diabetes mellitus, atherosclerosis, Alzheimer’s disease and cancer [21, 37]. Remarkably, the ER also plays an important role during immune responses to infection and malignancy. For example, during bacterial infection, Toll-like receptor (TLR) activation triggers splicing of *XBP1* mRNA, possibly in response to the increased biosynthesis of secreted inflammatory mediators, increasing the capacity for protein secretion and thus contributing to an augmented inflammatory response [38–40]. In addition, induction of GADD34 is required for cytokine expression during viral infection; however, in contrast to ER stress, pathogen-induced induction of GADD34 appears to be independent of CHOP [41, 42]. Nevertheless, sustained activation of the UPR can impair the immune response by triggering cell death [26, 43].

Previously, it has been shown that infection of airway epithelia or *Caenorhabditis elegans* with *P*. *aeruginosa* can elicit an UPR [39, 44, 45]. In worms, activation of the IRE1-XBP-1 branch of the UPR was dependent on p38 MAPK-signalling [39], but it is unknown if this signalling response is conserved in humans. Moreover, it is unclear whether living bacteria are required for the induction of ER stress or if unidentified secreted factors are sufficient.

In the present study, we set out to test the hypothesis that virulence factors secreted by *P*. *aeruginosa* trigger the UPR in human cells via the p38 MAPK pathway. We found that p38 MAPK signalling was required for the response of human epithelial cultures to bacterial conditioned medium and that the secreted factors pyocyanin and AprA contribute to the induction of ER stress. Furthermore, we showed that induction of the ISR target GADD34 is mediated by the iron-regulated kinase HRI and this induction protects the host against the toxic effects of *P*. *aeruginosa*.

## Results

### Conditioned medium of *P*. *aeruginosa* strain PAO1 causes ER stress in primary bronchial epithelial cells

**Fig 2:**
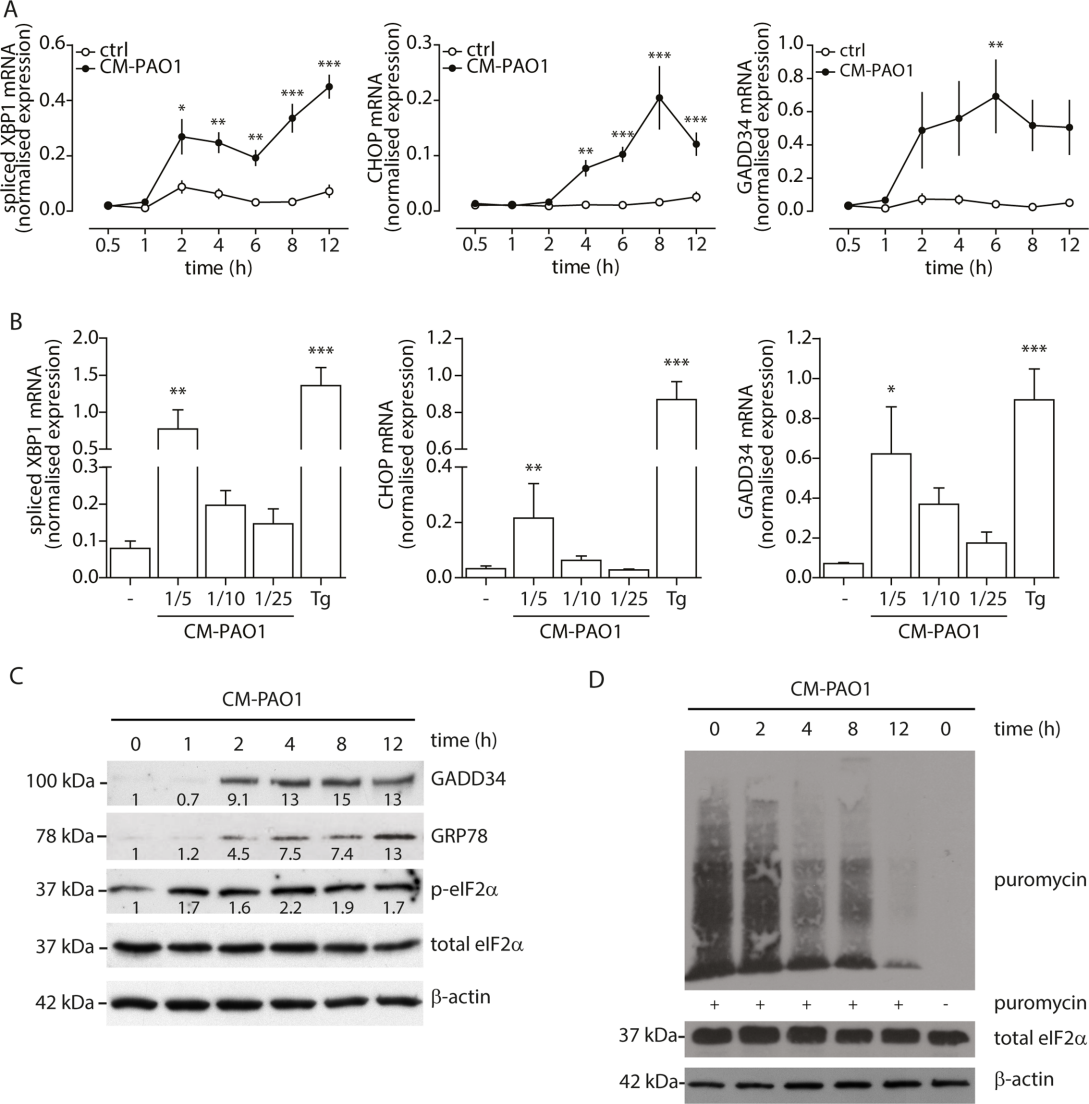
*P*. *aeruginosa* secreted virulence factors induce ER stress in primary bronchial epithelial cells. A. Time-dependent induction of ER stress in primary bronchial epithelial cells, as assessed by *XBP1* splicing, *CHOP* and *GADD34* mRNA after treatment with CM-PAO1 (n = 5; mean ± SEM). B. Dose-response of spliced *XBP1*, *CHOP* and *GADD34* mRNA in primary bronchial epithelial cells treated with CM-PAO1 for 12 hours (n = 5; mean ± SEM). C. Time-dependent phosphorylation of eIF2α (p-EIF2α) and synthesis of GADD34 and GRP78 (visualised with anti-KDEL antibody). Relative quantifications for each protein are shown within; representative of n = 3). D. Time-dependent decrease of puromycin incorporation in nascent proteins. Total eIF2α and β-actin serve as loading controls. * p<0.05, ** p<0.01, ***p<0.001 versus control (ctrl) with two-way repeated-measurements ANOVA (Bonferroni *post-hoc*) or untreated (-) with a one-way repeated-measurements ANOVA (Bonferroni *post-hoc*).

Infection with live *P*. *aeruginosa* has previously been shown to induce the UPR in mouse macrophages and human immortalized bronchial epithelial cells [40, 45]. To identify whether *P*. *aeruginosa* could induce the UPR in primary bronchial epithelial cells (PBEC) and whether living bacteria were necessary for this, we stimulated PBEC with filter-sterilised conditioned medium (CM) from *P*. *aeruginosa* strain PAO1 (CM-PAO1), containing secreted virulence factors without living bacteria. Treatment with CM-PAO1 induced ER stress in a time- and dose-dependent manner, as evidenced by a 9.9-fold increase of splicing of *XBP1* mRNA (p<0.01), a 12.8-fold increase of *CHOP* mRNA (p = 0.02) and a 16.2-fold increase of *GADD34* mRNA (p<0.05) after 8–12 hours (Fig 2A and 2B). This was accompanied by an increase in phosphorylation of eIF2α and protein expression of GADD34 and GRP78 (Fig 2C). This increase in phosphorylated eIF2α was accompanied by a decrease in global protein translation as assessed by puromycin incorporation in nascent proteins (Fig 2D) [46]. In line with previous reports [47–49], CM-PAO1 gradually impaired epithelial integrity until the monolayer was completely disrupted after 24 hours. Although the epithelial layer was disrupted by CM-PAO1 (as reported by trans-epithelial resistance; S1A Fig and visualised by light microscopy; S1B Fig), the cell membranes themselves remained intact as reported by exclusion of trypan blue stain (S1B Fig).

### Induction of ER stress in human bronchial epithelial cells by *P*. *aeruginosa* is dependent on p38 MAPK

**Fig 3:**
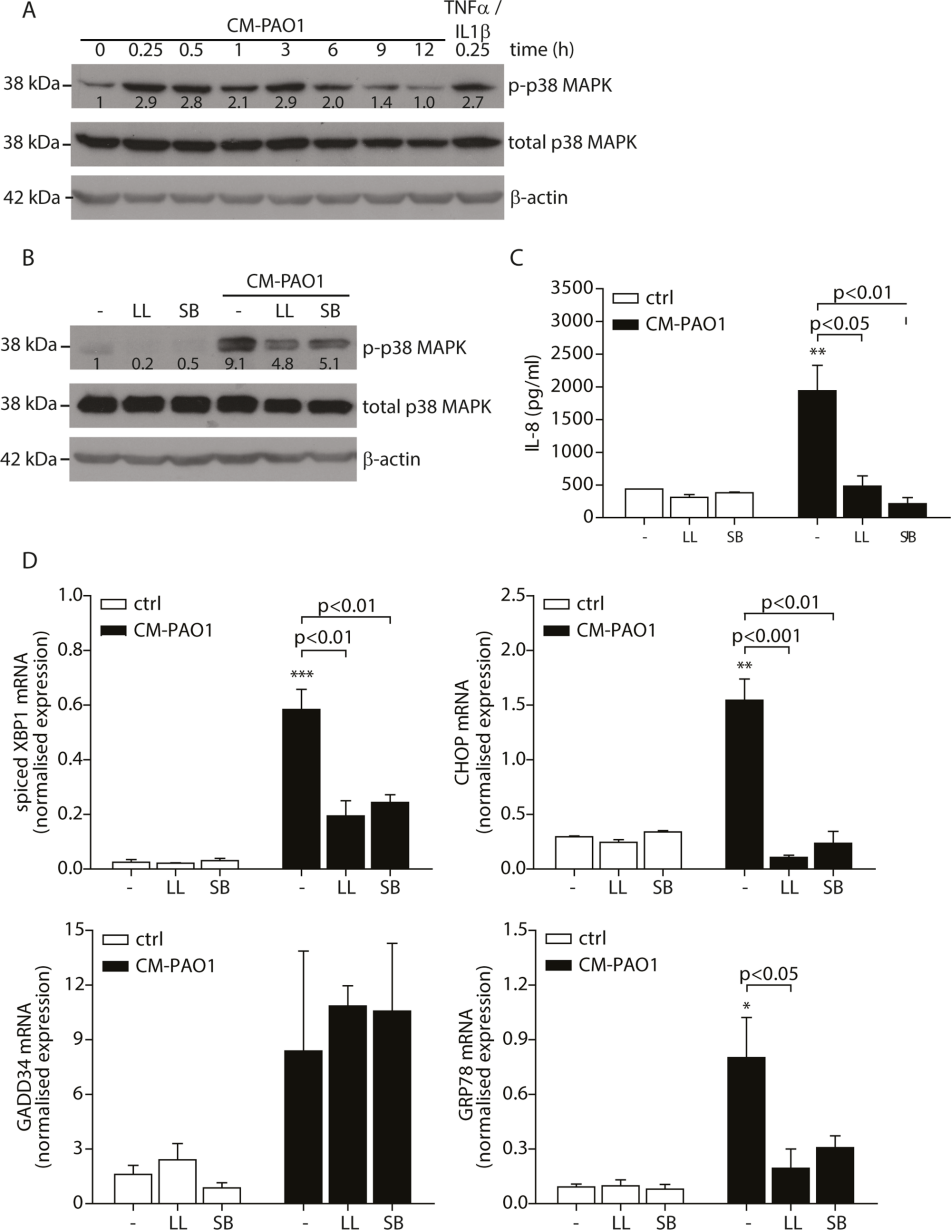
Conditioned medium of *P*. *aeruginosa* induces ER stress via TAK1-p38 MAP kinase (MAPK). A. Time-dependent phosphorylation of p38 MAPK in 16HBE after treatment with CM-PAO1. Total p38 MAPK and β-actin serve as loading controls. Numbers display the fold increase of phosphorylated p38 MAPK to total p38 MAPK compared to phosphorylated p38 MAPK at t = 0 (representative of n = 3). B. Western blot of phosphorylated p38 MAPK from 16HBE after pre-treatment for 30 min with the TAK-1 inhibitor LL-Z1640-2 (LL) or p38 MAPK inhibitor SB203580 (SB), followed by CM-PAO1 stimulation for 6 hours. Total p38 MAPK and β-actin serve as loading controls. Numbers display the fold increase of phosphorylated p38 MAPK to total p38 MAPK compared to phosphorylated p38 MAPK at t = 0 (representative of n = 3). C. IL-8 release of 16HBE cells treated as in B (n = 3; mean ± SEM). D. Normalised mRNA levels of *spliced XBP1*, *CHOP*, *GADD34* and *GRP78* in 16HBE cells, treated as B (n = 3; mean ± SEM). All values are normalised to the housekeeping genes *RPL13A* and *ATP5B*. * p<0.05, ** p<0.01, ***p<0.001 versus untreated (-) with a one-way repeated-measurements ANOVA (Bonferroni *post-hoc*).

Infection of *C*. *elegans* with *P*. *aeruginosa* has been reported to cause splicing of *XBP1* mRNA in a p38 MAPK-dependent manner [39]. To exclude the effects of donor variation and complex nutrient/growth factor requirement of primary cells, we tested whether exposure of 16HBE cells, a SV-40 transformed bronchial epithelial cell line, to *P*. *aeruginosa* conditioned medium would trigger phosphorylation of p38 MAPK and activate the UPR. We observed that CM-PAO1 caused prolonged phosphorylation of p38 MAPK in 16HBE cells up to 6 hours (Fig 3A). We reasoned that the activation of p38 MAPK after 15 minutes might represent the activation of TLR signalling, since stimulation of HEK-TLR2 or HEK-TLR4 cells [50] with CM-PAO1 demonstrated robust TLR2 and TLR4 activation. The sustained activation was similar to that observed in *C*. *elegans* infected with *Pseudomonas* [39], which suggests the importance of p38 MAPK in the induction of the UPR. To examine if p38 MAPK signalling was required for the ER stress response, we pre-treated 16HBE cells with an inhibitor of p38 MAPK (SB203580) or an inhibitor of TAK1 (5Z-7-oxozeanol, better known as LL-Z1640-2), a kinase upstream of p38 MAPK. We then exposed cells to CM-PAO1 and observed that both compounds markedly reduced activation of p38 by CM-PAO1 (Fig 3B). In addition, both compounds reduced secretion of IL-8 in response to CM-PAO1 treatment (Fig 3C). Of note, these compounds strongly inhibited splicing of *XBP1* mRNA and abrogated the induction of *CHOP* and *GRP78* mRNA (Fig 3D). However, the induction of *GADD34* was insensitive to the inhibitors (Fig 3D) suggesting the involvement of an additional pathway independent of CHOP.

### Pyocyanin is able to induce ER stress

**Fig 4:**
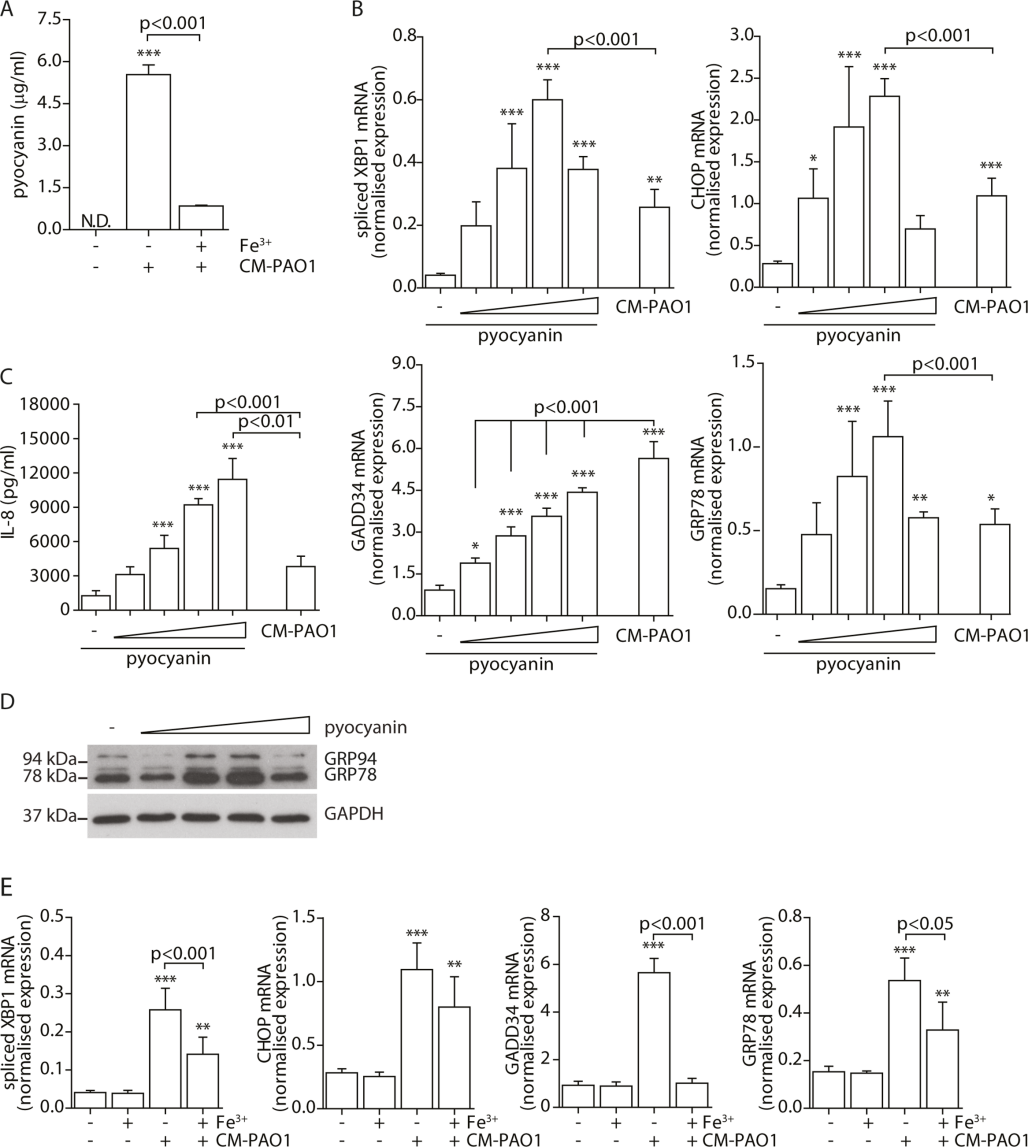
Pyocyanin is able to cause ER stress. A. Quantitation of pyocyanin in CM-PAO1. Iron (Fe^3+^) was supplemented in the culture medium of the bacteria to inhibit virulence factor secretion (n = 3; mean ± SEM). B. Normalised mRNA expression levels of *spliced XBP1*, *CHOP*, *GADD34* and *GRP78* after pyocyanin treatment (0-1-3-10-30 μM) (n = 3; mean ± SEM). All values are normalised to the housekeeping genes *RPL13A* and *ATP5B*. C. IL-8 release of 16HBE cells treated as in B (n = 3; mean ± SEM). D. Western blot for GRP78/GRP94 and GAPDH (loading control) from 16HBE cell lysates treated as in B. (representative of n = 3). E. Spliced *XBP1*, *CHOP*, *GADD34* and *GRP78* mRNA levels in 16HBE cells exposed to CM-PAO1 derived after growth in the presence or absence of iron (Fe^3+^) (n = 3; mean ± SEM). All values are normalised to the housekeeping genes *RPL13A* and *ATP5B*. * p<0.05, ** p<0.01, ***p<0.001 versus untreated (-) with a one-way repeated-measurements ANOVA (Bonferroni *post-hoc*).

To prepare *P*. *aeruginosa* conditioned medium, cultures were grown for 5 days (see Experimental procedures and [47]) to a high optical density, at which quorum-sensing is activated in this strain, thus triggering the production of a variety of virulence factors among which the cytotoxic exoproduct pyocyanin. When pyocyanin levels in CM-PAO1 were measured, values up to 5.5 μg/ml (26 μM) were detected (Fig 4A), which were similar to values observed in sputum of CF patients colonised with *P*. *aeruginosa* [51]. We first wished to determine if pyocyanin was an important mediator of the observed ER stress response by CM-PAO1. To this end, *P*. *aeruginosa* bacterial cultures were supplemented with iron to suppress pyocyanin production together with other iron-regulated factors (Fig 4A). The conditioned medium prepared in this manner was significantly less efficient at triggering the splicing of *XBP1* mRNA and at increasing expression of *GRP78* mRNA (Fig 4B). Surprisingly, *CHOP* mRNA was not significantly affected (Fig 4B), whereas *GADD34* mRNA induction was completely abrogated.

These experiments provided only indirect support for the involvement of pyocyanin, since iron supplementation also affects production of other *P*. *aeruginosa* virulence factors and may also affect host cells. We therefore tested whether purified pyocyanin could induce ER stress in 16HBE cells. Treatment with purified pyocyanin caused dose-dependent splicing of *XBP1* mRNA, induction of *CHOP* and *GRP78* mRNAs and expression of GRP78 and GRP94 protein (Fig 4C and 4D), maximal at 10 μM (2.1 μg/ml). In contrast, *GADD34* mRNA continued to rise up to a maximum at ≥ 30 μM (6.3 μg/ml) of pyocyanin (Fig 4D). Once again, this suggested that induction of *GADD34* in this system might not simply reflect activation by ER stress. As expected, pyocyanin potently induced secretion of IL-8 by 16HBE cells (Fig 4E) [10].

Since pyocyanin is a redox active toxin, we tested the effect of co-administration of the anti-oxidants N-acetylcysteine (10 mM) and glutathione reduced ethyl-ester (10 mM) for 24 hours. Both failed to ameliorate the ER stress response suggesting that pyocyanin caused ER dysfunction independent of causing oxidative stress [52, 53] (see online repository).

Taken together, these observations suggested that conditioned medium of *P*. *aeruginosa* caused ER stress via multiple virulence factors, including pyocyanin. Furthermore, the induction of GADD34 appeared to involve an additional pathway independent of CHOP.

### Identifying other factors mediating ER stress

**Fig 5:**
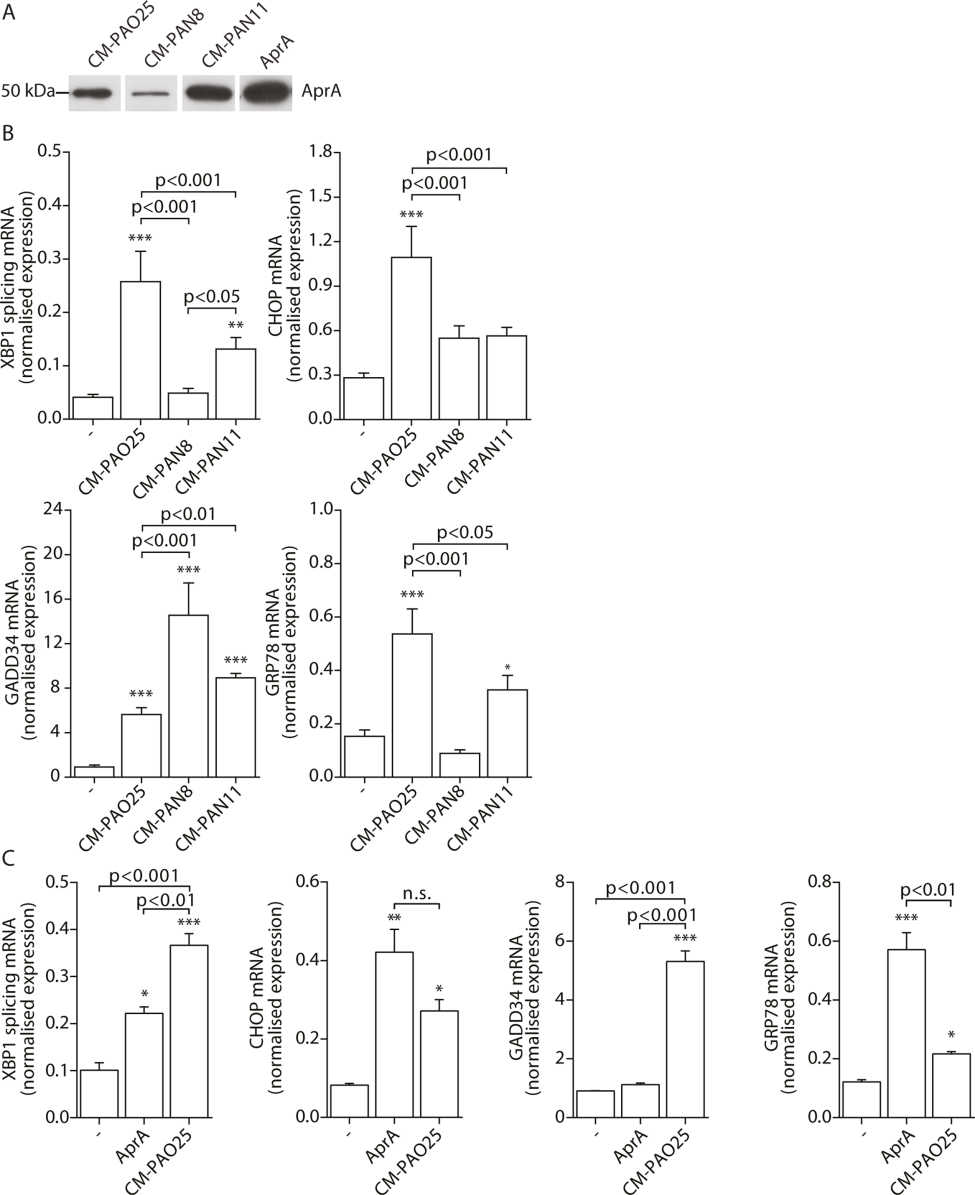
The *P*. *aeruginosa* secreted virulence factors alkaline protease (AprA) and pyocyanin mediate ER stress. A. Western blot analysis of conditioned medium (CM) of strains PAO25, PAN8 and PAN11 for AprA (representative of n = 2; complete blot is shown in S3B Fig). Equal volumes of the conditioned medium were loaded onto the gel. AprA displays 200 nM purified AprA and serves as a positive control. B. Normalised expression levels of *XBP1* splicing, *CHOP*, *GADD34* and *GRP78* mRNA in 16HBE cells after stimulation with CM-PAO25, CM-PAN8 or CM-PAN11 (n = 3; mean ± SEM). All values are normalised to the housekeeping genes *RPL13A* and *ATP5B*. C. Normalised expression values of *spliced XBP1*, *CHOP*, *GADD34* and *GRP78* mRNA in 16HBE cells after stimulation with 10 nM purified AprA. All values are normalised to the housekeeping genes *RPL13A* and *ATP5B*. * p<0.05, ** p<0.01, ***p<0.001 versus untreated (-) with a one-way repeated-measurements ANOVA (Bonferroni *post-hoc*).

Having found evidence for the involvement of multiple virulence mechanisms in the induction of ER stress, we next attempted to determine their identities. The *P*. *aeruginosa* AB toxin exotoxin A is known to cause translational attenuation by catalysing the ADP-ribosylation of elongation factor 2 (EF2) [54]. We investigated whether purified exotoxin A could also induce ER stress, but detected no increase in *spliced XBP1*, *CHOP*, *GADD34* or *GRP78* mRNA (S2A Fig) nor the phosphorylation of eIF2α (S2B Fig). Next, to more broadly explore the involvement of other potential virulence factors, we made use of strains of *P*. *aeruginosa* that lacked specific toxic products: PAN8, a *lasB aprE* double mutant, which is deficient in the production of elastase [55] and the secretion of AprA; PAN11, an *xcpR lasB* mutant, which is deficient in the production of elastase and the secretion of all other substrates of the type II protein secretion system but still produces AprA; and PAO25, a *leu arg* double mutant derivative of PAO1 and the direct parental strain of both mutants (Table 1). CM-PAO25 did not differ from CM-PAO1 in the content of all toxins measured (S3A and S3B Fig) and in inducing *spliced XBP1*, *CHOP*, *GADD34* and *GRP78* mRNA (S3C Fig). In spite of the *aprE* mutation, still traces of AprA were detected in the culture supernatant of the PAN8 strain (Fig 5A), presumably due to cell lysis during the 5-days growth period.

**Table 1:** Bacterial strains.

Strain	Characteristics	Ref.
PAO1	Wild-type	ATCC; BAA-47
PAO25	PAO1 *leu arg*	[70]
PAN8	PAO25 lasB*::km^R^ aprE::ΩHg*	[55]
PAN11	PAO25 *xcpR-54 lasB::km^R^*	[55]

When 16HBE cells were incubated with CM-PAN8 (lacking elastase and AprA), *XBP1* mRNA splicing and induction of *GRP78* mRNA were completely abolished, and only low induction of *CHOP* mRNA remained (Fig 5B). In contrast, the response of 16HBE cells to CM-PAN11 (containing AprA, but no elastase or other substrates of the type 2 secretion system) was much less affected relative to CM-PAO1 treatment (Fig 5B), indicating that the reduced response to CM-PAN8 is primarily due to the absence of AprA in this CM rather than to the absence of elastase. Indeed, stimulating 16HBE cells with purified elastase did not elicit an ER stress response within 24 hours (see online repository). On the other hand, incubation with 10 nM purified AprA induced the splicing of *XBP1* mRNA, and up-regulated *CHOP* and *GRP78* mRNA (Fig 5C). These experiments suggested that, in addition to pyocyanin, AprA also contributed to the induction of ER stress in 16HBE cells. We therefore next generated conditioned medium of a series of specific AprA and pyocyanin mutant strains to demonstrate the relative contribution of AprA and pyocyanin to the induction of ER stress. However, these experiments were inconclusive because the corresponding wild type strains did not induce sufficient ER stress (see online repository).

Remarkably, once again the induction of *GADD34* mRNA followed a distinct trend from the other markers of ER stress. Particularly a lack of AprA (in CM-PAN8) was correlated with an increased expression of *GADD34* (Fig 5B), whilst purified AprA did not induce GADD34 mRNA (Fig 5C). This suggested that an unrelated mechanism regulated *GADD34* induction by CM-PAO1 and that this might be independent of ER stress.

### GADD34 is regulated via the integrated stress response (ISR) independent of PERK

**Fig 6:**
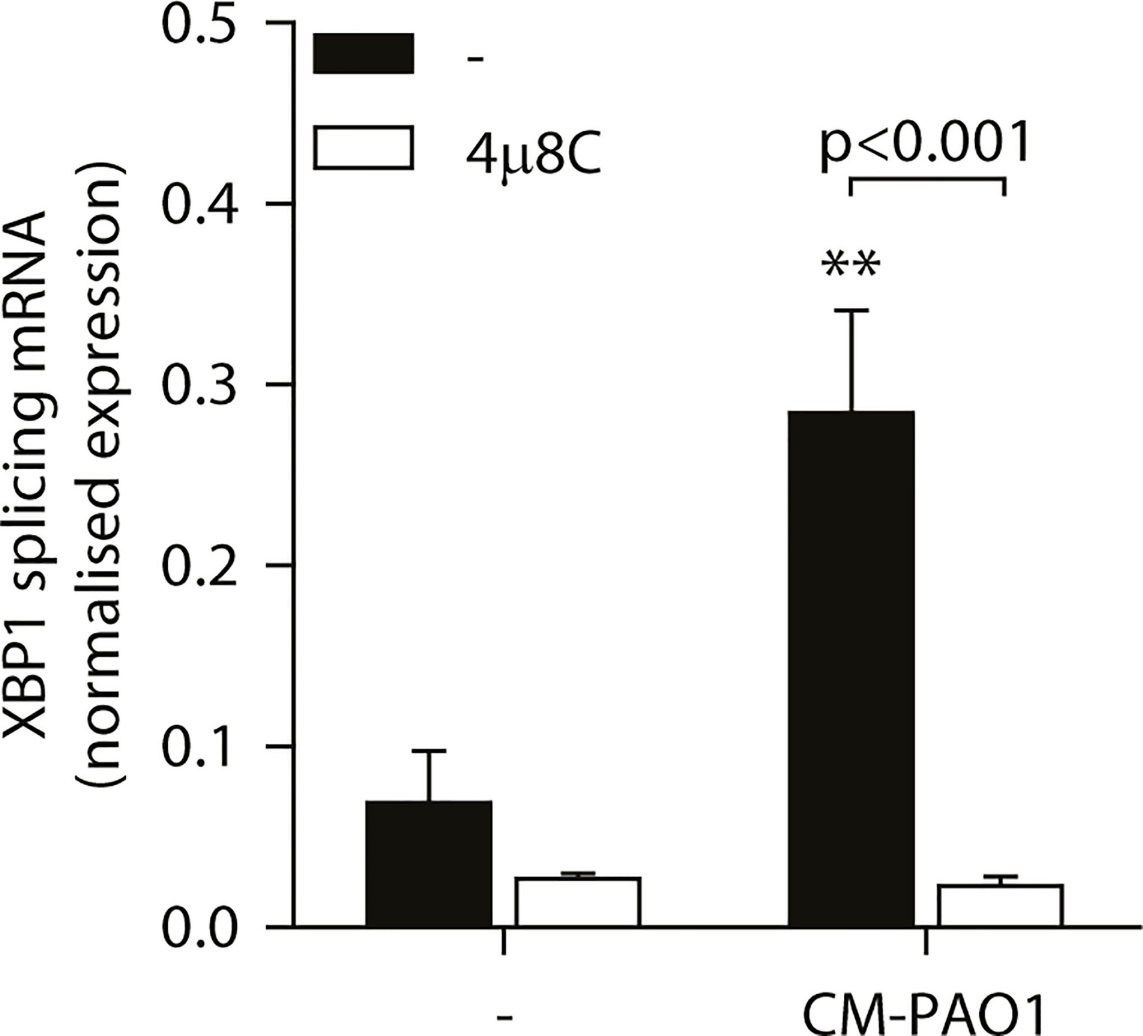
Splicing of XBP1 mRNA is dependent on the ER stress responsive kinase IRE1α. Splicing of *XBP1* mRNA in 16HBE cells after treatment with CM-PAO1 in the presence of 30 μM 4μ8C, a selective inhibitor of the ER stress responsive kinase IRE1α (n = 3; mean ± SEM). All values are normalised to the housekeeping genes *RPL13A* and *ATP5B*. * p<0.05, ** p<0.01, *** p<0.001 versus untreated (-) with a two-way repeated-measurements ANOVA (Bonferroni *post-hoc*).

To examine the involvement of ER stress-dependent and-independent responses to CM-PAO1, we next made use of the specific inhibitor of IRE1, 4μ8C, which blocks splicing of *XBP1* mRNA during ER stress ([56] and Fig 6). Of note, this compound not only attenuated the splicing of *XBP1* mRNA elicited by CM-PAO1, but interestingly, it also attenuated the secretion of IL-8 by 16HBE in response to CM-PAO1 (S4A Fig).

**Fig 7:**
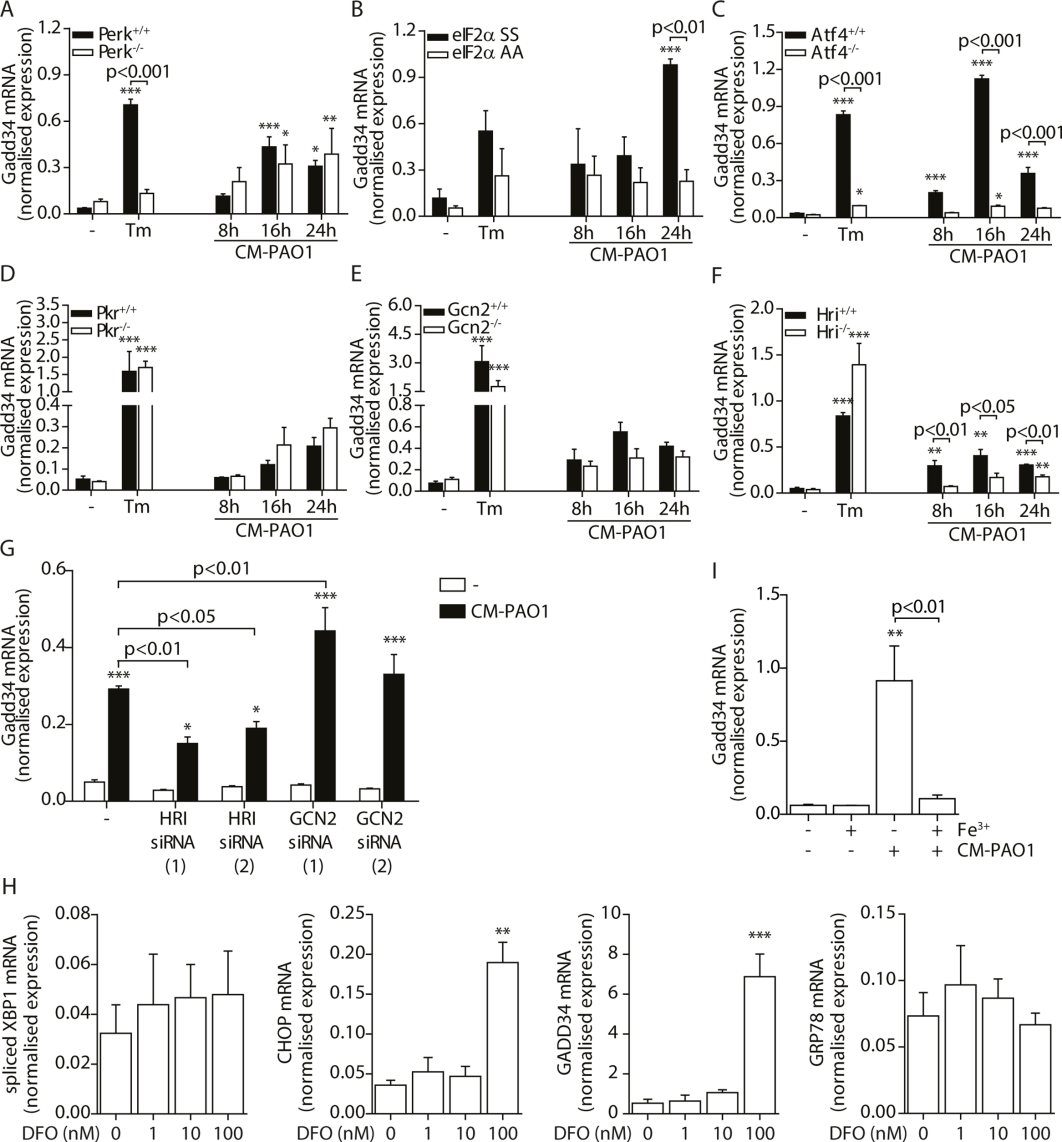
*GADD34* mRNA is regulated via the activation of the integrated stress response by *P*. *aeruginosa*. B-G. *Gadd34* mRNA normalised expression in *Perk* ^*-/-*^, *eIF2α* ^*AA*^, *Atf4* ^*-/-*^, *Pkr* ^*-/-*^, *Gcn2* ^*-/-*^ and *Hri* ^*-/-*^ mouse embryonic fibroblasts (MEFs) exposed to CM-PAO1 for 8, 16 or 24 hours or tunicamycin (Tm) for 6 hours as a positive control (n = 3; mean ± SEM). All values are normalised to the housekeeping genes *Actb* and *Sdha*. H. *GADD34* mRNA levels in HeLa cells upon exposure to CM-PAO1 after knock-down of GCN2 or HRI with siRNA (n = 3; mean ± SEM). All values are normalised to the housekeeping genes *RPL13A* and *ATP5B*. I. Normalised expression values of *spliced XBP1*, *CHOP*, *GADD34* and *GRP78* mRNA in 16HBE cells after stimulation with 1–100 nM deferoxamine (DFO). All values are normalised to the housekeeping genes *RPL13A* and *ATP5B*. J. *Gadd34* mRNA levels in wild-type MEFs after repletion of the cell culture medium with iron (Fe^3+^) when treated with CM-PAO1 (n = 3; mean ± SEM). The first column (- Fe^3+^,—CM-PAO1) reflects medium control cells, without adding or depleting iron from the cell culture medium. All values are normalised to the housekeeping genes *Actb* and *Sdha*. * p<0.05, ** p<0.01, *** p<0.001 versus untreated (-) with a two-way repeated-measurements ANOVA (Bonferroni *post-hoc*).

During ER stress, the kinase PERK phosphorylates eIF2α, thereby activating the ISR. When *Perk* ^*-/-*^ mouse embryonic fibroblasts (MEFs) were exposed to CM-PAO1, the induction of *Gadd34* mRNA was unaffected, while the response to the ER stress-inducing agent tunicamycin (Tm) was abrogated (Fig 7A). However, phosphorylation of eIF2α was required for the induction of *Gadd34* mRNA in response to CM-PAO1 as demonstrated by the failure of the conditioned medium to induce *Gadd34* mRNA in fibroblasts homozygous for the eIF2α^AA^ mutation, which renders them insensitive to all eIF2α kinases (Fig 7B). Moreover, ATF4, a transcription factor translationally up-regulated upon phosphorylation of eIF2α, was essential for the induction *Gadd34* mRNA by CM-PAO1 (Fig 7C). As we have shown previously [26], CHOP was only partially required for tunicamycin (ER stress)-induced expression of *Gadd34* mRNA (S4B Fig). The same was observed for CM-PAO1, although it did not reach statistical significance (S4B Fig). Interestingly, murine fibroblasts stimulated with CM-PAO1 failed to splice *Xbp1* mRNA (S4C Fig), suggesting that activation of IRE1 by CM-PAO1 may be less important in this cell type than in human epithelial cells. However, reassuringly, ISR-dependent signalling in response to pseudomonal toxins was preserved in these cells and, once again, expression of *Chop* mRNA was regulated via eIF2α and ATF4. As had been observed for *Gadd34*, *Chop* induction was independent of PERK, suggesting that in MEFs treated with CM-PAO1, *Chop* was induced by a stimulus other than ER stress (S4D–S4F Fig).

We next examined which eIF2α kinase was responsible for activation of the ISR by CM-PAO1. To this end, we made use of *Pkr* ^*-/-*^, *Gcn2* ^*-/-*^ and *Hri* ^*-/-*^ MEFs [25, 57, 58] and observed a significant deficit of CM-PAO1 induction of *Chop* and *Gadd34* mRNA in *Hri* ^*-/-*^ cells, suggesting the involvement of the iron-sensing kinase HRI (Fig 7D–7F and S4G–S4I Fig). In contrast, although it has been suggested previously that GCN2 is involved in the stress response induced by *P*. *aeruginosa* in gut epithelial cells [59], we observed no significant effect on the induction of *Gadd34* mRNA in *Gcn2* ^*-/-*^ cells (Fig 7E). We therefore went on to deplete either GCN2 or HRI in HeLa cells using two separate siRNA oligonucleotides for each gene and obtained similar results: whereas both siRNAs directed against HRI decreased induction of *Gadd34* mRNA, one siRNA directed against GCN2 had no effect whereas the other even increased *Gadd34* mRNA expression (Fig 7H and S4J Fig). Whereas we cannot exclude the possibility that this increasing effect of one siRNA directed against GCN2 may result from putative off-target effects, we conclude that these data support a role for HRI rather than GCN2.

Since RPMI is an iron-poor medium, we reasoned that the CM-PAO1 would limit iron availability to epithelial cells, e.g. by the presence of siderophores [60], which might activate HRI through depletion of iron from the culture medium. We therefore first evaluated the effect of iron depletion of the epithelial cell culture medium using deferoxamine (DFO). DFO treatment resulted in a marked increase in the expression of the ISR and UPR related genes CHOP and GADD34, whereas GRP78 and spliced XBP1 were not affected (Fig 7H). This is line with selective activation of the ISR by iron depletion. We next confirmed the presence of the iron-chelating siderophore pyoverdine in the CM-PAO1 by the bright fluorescence of the medium upon exposure to UV light (see online repository). To test the possible involvement of iron depletion in CM-PAO1-mediated *Gadd34* induction, we supplemented the epithelial cell culture medium with iron, which indeed completely suppressed the induction of *Gadd34* mRNA (Fig 7I and S4K Fig).

Taken together, these data demonstrate that CM-PAO1 induces splicing of *XBP1* mRNA (ER stress) in human bronchial epithelial cells, while induction of *GADD34* predominantly reflects an iron-dependent ISR mediated by the eIF2α kinase HRI.

### The role of Gadd34 induction in cell survival

**Fig 8:**
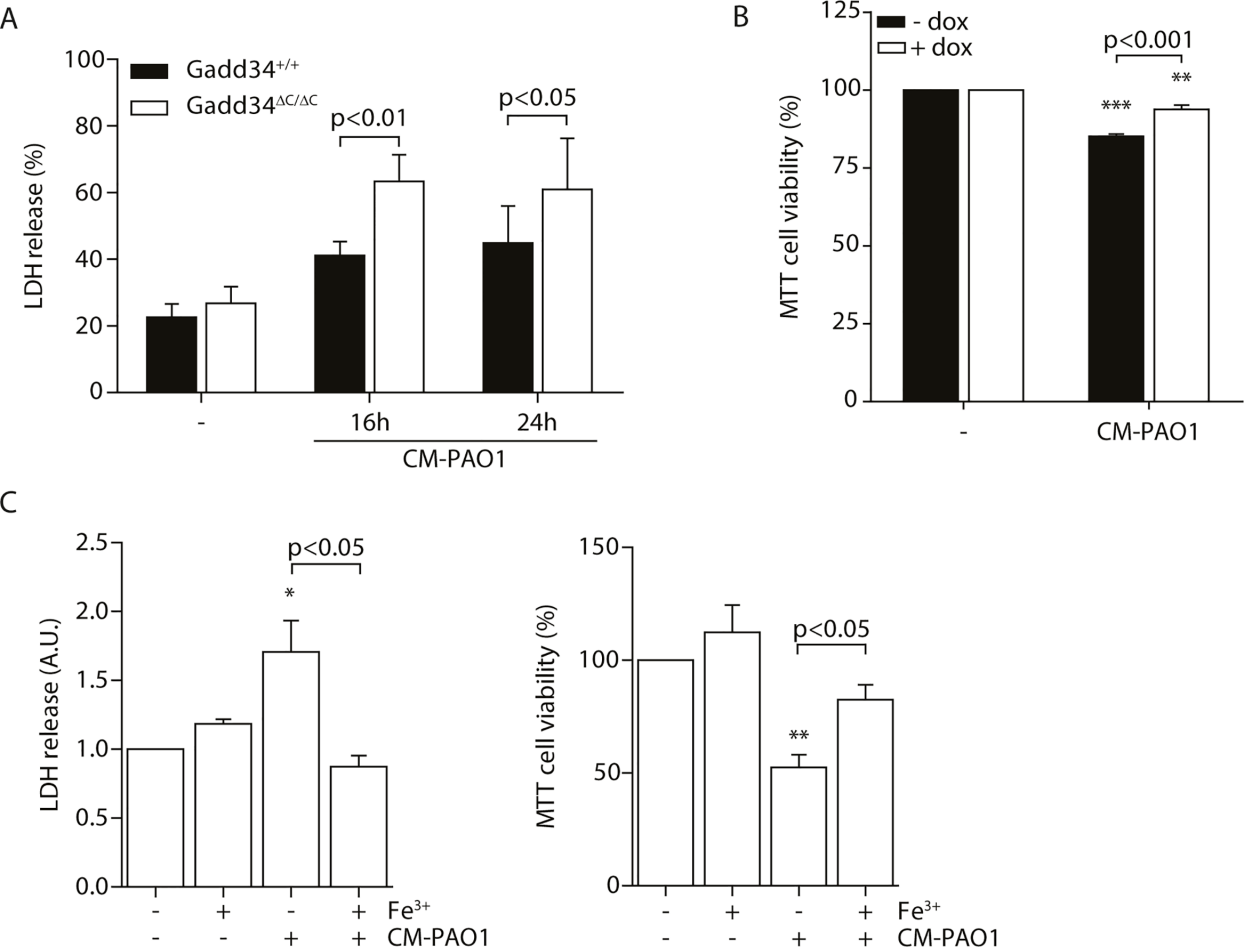
Induction of GADD34 protects against *P*. *aeruginosa* mediated cell cytotoxicity. A. LDH release of *Gadd34* ^*+/+*^ and *Gadd34* ^*ΔC/ΔC*^ MEFs after stimulation with CM-PAO1 for 16 and 24 hours (n = 3; mean ± SEM). B. MTT assay assessing cell viability of HeLa cells conditionally expressing GADD34 (± dox) after treatment with CM-PAO1 (n = 3; mean ± SEM). C. LDH release (*left*) and cell viability assessed with a MTT assay (*right*) of wild-type MEFs treated with CM-PAO1 after repleting the cell culture medium with iron (Fe^3+^) (n = 3; mean ± SEM). * p<0.05, ** p<0.01, *** p<0.001 versus untreated (-) with a two-way repeated-measurements ANOVA (Bonferroni *post-hoc*).

During chronic ER stress in cell and animal models of disease, the induction of GADD34 appears to mediate cellular toxicity [26, 43]. In contrast, during the acute stress of SERCA pump inhibition by thapsigargin, GADD34 has been shown to be protective [61]. To test the role of ER stress-independent induction of GADD34 by exposure to CM-PAO1, we made use of *Gadd34* ^*ΔC/ΔC*^ MEFs [61], which lack GADD34 phosphatase activity. Cells expressing wild-type GADD34 were more resistant to the cytotoxic effects of CM-PAO1 compared with *Gadd34* ^*ΔC/ΔC*^ fibroblasts, as reported by the release of lactate dehydrogenase (LDH) (Fig 8A). To confirm these findings, we repeated these experiments in HeLa cells expressing GADD34 from a tetracycline-responsive promoter. The induction of GADD34 with doxycycline significantly increased cell viability upon exposure to CM-PAO1 (Fig 8B). When the cell culture medium of wild-type cells was supplemented with iron, the release of LDH was prevented (Fig 8C, *left panel*). Iron supplementation was also observed to rescue cell viability reported by MTT assay (Fig 8C, *right panel*).

Taken together, these data suggest that the toxicity of CM-PAO1 is sensitive to iron and that HRI-mediated induction of GADD34 is protective in this context. Supplementation with iron relieves both the cytotoxicity and the requirement for induction of GADD34.

## Discussion

It is known that a normal response to ER stress is required for an efficient innate immune response to bacterial infection [39], but whether live bacteria are required for this has been unclear. In this study, we have shown that secreted virulence factors of *P*. *aeruginosa* cause ER stress in primary bronchial epithelial cells and in a cell line, and that this is mediated by TAK1 and phosphorylated p38 MAPK. In addition, we have identified GADD34 induction via an ER-stress independent ISR. We have demonstrated pyocyanin to be one of the factors eliciting these responses, while AprA contributes to the activation of the UPR. We were however unable to establish the relative contribution of pyocyanin and AprA to the activation of the UPR. In contrast, activation of the ISR with induction of *GADD34* mRNA is most likely a response to reduced iron availability and may serve a cytoprotective role during exposure to conditioned medium of *P*. *aeruginosa*.

In line with these observations, phosphorylation of p38 MAPK has previously been shown to be involved in the splicing of *XBP1* upon infection with *P*. *aeruginosa* [39, 45], although the involvement of TAK1 upstream of p38 MAPK and its essential involvement in the activation of CHOP and GRP78 are novel findings. Interestingly, GADD34, classically a downstream target of CHOP, was regulated independently of the TAK1-p38 MAPK pathway. The induction of GADD34 is only partially dependent on CHOP (S4B Fig and [26]), but it is absolutely reliant on phosphorylation of eIF2α and ATF4 [26]. This is concordant with the recent description of a virus-induced “microbial stress response” mediated via the PKR/eIF2α/ATF4 pathway, which fails to induce CHOP, but potently induces GADD34 [41, 42].

In contrast to the response of human airway epithelial cells, *P*. *aeruginosa* conditioned medium failed to cause splicing of *Xbp1* mRNA in murine fibroblasts, suggesting that ER stress may not be a conserved feature of the cellular response to this insult. This is unsurprising, as induction of ER stress is known to be highly cell-type dependent [40]. In the absence of ER stress in the murine fibroblasts, the induction of *Chop* and *Gadd34* suggests that activation of the ISR by the secreted virulence factors may be a more conserved response. Of note, in human bronchial epithelial cells, the induction of CHOP seems primarily subordinate to an ER stress-induced ISR, rather than the microbial stress response (S7 Fig). Consequently, induction of *CHOP* was dependent on the TAK1-p38 MAPK pathway in those cells (Fig 3D) and its induction was only partially inhibited when bacterial cultures were supplemental with iron (Fig 4B), in contrast to MEFs where *Chop* induction was dependent on HRI (S4I Fig).

Recent evidence suggests that bacterial components may function as triggers for the UPR. Flagellin has been shown to induce an atypical ER stress response in CF bronchial epithelial cells during live infection [45], while N-(3-oxo-dodecanoyl) homoserine lactone (C12) has been observed to phosphorylate eIF2α and activate p38 MAPK [62]. We have now shown that at least two secreted virulence factors, pyocyanin and AprA, also contribute to this ER stress response to *Pseudomonas*. More research has to be done to assess the involvement of (other) individual virulence factors.

High concentrations of pyocyanin also mediated an ER stress-independent, ISR-dependent induction of GADD34 (Fig 4E). We were able to identify a crucial role for iron availability and for the iron-sensing kinase HRI in this response, although we cannot fully exclude a role for the kinase GCN2 that has been previously implicated in responses to Pseudomonas spp [59]. Of note, it is possible that the protective effect of GADD34 is unrelated to its ability to dephosphorylate p-eIF2alpha. Interestingly, AprA was not involved in the induction of the ISR response but rather appeared to dampen it, since considerably higher GADD34 expression was observed when conditioned medium of the *aprE* mutant PAN8 was used to stimulate the cells (Fig 5B). Among other possibilities, an explanation for this observation could be that AprA present in the conditioned medium of the wild-type strain partially degrades HRI, a possibility that warrants further investigations. The discovery of this ER-independent ISR may plausibly offer novel potential therapeutic targets.

It has been shown recently that spliced XBP1 is required for C12-mediated apoptosis [62]. Remarkably, exposure of cells to C12 does not itself trigger the splicing of *XBP1* mRNA suggesting that basal levels of XBP1 splicing are both necessary and sufficient for this response. Moreover, the transcriptional activity of spliced XBP1 does not appear to be required for this cell death, indicating that the spliced XBP1 protein may have additional, as yet unidentified, activities. C12 appears able to trigger the ISR in an ER stress-independent matter, although the mechanism for this remains to be determined. It would be interesting to determine if C12 can activate HRI.

Chronic elevation of GADD34 in ER stress can mediate cellular toxicity [26], but GADD34 has been shown to be protective during the acute stress of SERCA pump inhibition with thapsigargin, which depletes the ER of calcium [61]. As with thapsigargin, *P*. *aeruginosa* has been associated with altered ER calcium signalling [38, 44]. It is therefore of interest that expression of GADD34 reduced cell toxicity and increased cell survival upon iron deficiency caused by treatment with conditioned medium from *P*. *aeruginosa*. It has been shown that lungs of cystic fibrosis patients lack the ability to induce GADD34 [45], which might plausibly lead to increased cytotoxicity or altered innate immunity due to *Pseudomonas* infection of the lungs of CF patients. However, future *in vivo* studies are required to confirm the observed cytoprotective effect of GADD34 induction during *Pseudomonas* infections.

In summary, secreted virulence factors of the PAO1 strain of *P*. *aeruginosa*, including pyocyanin and AprA, are sufficient to elicit an ER stress response but the relative contribution of these virulence factors remains to be investigated. In contrast to these virulence factors, our findings strongly suggest that iron depletion mediated by the secretion of siderophores causes an ER stress-independent ISR. The induction of GADD34 by this may serve to ameliorate the toxic effects of *P*. *aeruginosa* conditioned medium.

## Materials and Methods

### Bacterial strains and preparation of conditioned medium of *P*. *aeruginosa*

All strains used in this study are listed in Table 1. CM was prepared as described previously with slight modifications [47]. Briefly, overnight bacterial cultures in Luria Broth were inoculated 1:50 into RPMI 1640 (Gibco, Life Technologies, Breda, the Netherlands) and incubated at 37°C shaking at 200 rpm. After 5 days, the cultures were centrifuged and supernatants were filter-sterilized through 0.22 μm pore-size filter (Whatman, Dassel, Germany) to obtain CM. Pyocyanin and AprA levels in CM were measured as described previously [63, 64].

### Cell culture

PBEC were obtained from tumour-free resected lung tissue by enzymatic digestion as described previously [65]. 16HBE cells (passage 4–15; kindly provided by Dr. D.C. Gruenert, University of California, San Francisco, CA, USA) were cultured in MEM (Invitrogen) supplemented with 1 mM HEPES (Invitrogen), 10% (v/v) heat-inactivated FCS (Bodinco, Alkmaar, the Netherlands), 2 mM L-glutamine, 100 U/ml penicillin and 100 μg/ml streptomycin (all from BioWhittaker). All MEFs were maintained as described previously [23, 26, 36, 66, 67]. HEK-TLR2 and HEK-TLR4 [50] were a kind gift from Prof. Dr. M. Yazdanbakhsh (Leiden University Medical Center, the Netherlands). HeLa cells were transfected for 6 hours with two different ON-TARGETplus Human EIF2AK1 siRNA (GCACAAACUUCACGUUACU and GAUUAAGGGUGCAACUAAA) and knockdown was assessed 48 hours after transfection (S5 Fig).

GADD34-N1-eGFP (kind gift form S. Shenolikar, Duke-NUS Graduate Medical School Singapore, Singapore) was excised with BglII and NotI and ligated into pTRE2-hyg plasmid (Clontech Laboratories, Mountain View, CA, USA) digested with BamHI and NotI. HeLa Tet-On advanced cells (Clontech Laboratories) were transfected with the pTRE2-hyg_GADD34-eGFP plasmid and selected with 600 μM hygromycin to generate a stable cell line conditionally expressing GADD34-GFP (S6 Fig). Positive cell clones were visualised by GFP expression in response to 1 μg/ml of doxycycline. Once identified, expanded and characterized, these clones were maintained in DMEM (Sigma) supplemented with 10% FBS and antibiotics (100 U/ml penicillin G, 100 μg/ml streptomycin, 200 μg/ml G418 and 200 μM hygromycin). Expression of GADD34 was typically induced using 1 μg/ml doxycycline (Sigma) for 24 hours.

Cells were exposed at 80–90% confluence for 24 hours (unless stated otherwise) to CM-PAO1 (1 in 5 dilution, unless stated otherwise), pyocyanin (1–30 μM), ammonium iron (III) citrate (100 μM; Fe^3+^), exotoxin A (1–10 ng/ml), AprA (10 nM), elastase (16–64 μg/ml) and/or DFO (1–100 nM) as indicated (all from Sigma). Puromycin (10 μg/ml; Sigma) was added 30 minutes before harvesting. Thapsigargin (100 nM; Sigma), TNFα and IL-1β (both 20 ng/ml; Peprotech, Rocky Hill, NJ) were used as positive controls. The compounds SB203580 (10 nM; Sigma) and 5Z-7-oxozeanol (also called LL-Z1640-2; 100 nM; TebuBio, Heerhugowaard, the Netherlands) were added 30 minutes before stimulation for the inhibition of p38 MAPK and TAK1, respectively. The specific IRE1-inhibitor 4μ8C (30 μM) [56] was a kind gift from Prof. Dr. D. Ron, University of Cambridge.

### Western blot

Cells were lysed in buffer H (10 mM HEPES, pH 7.9, 50 mM NaCl, 500 mM sucrose, 0.1 mM EDTA, 0.5% (v/v) Triton X-100, 1 mM PMSF, 1X Complete protease inhibitor cocktail (Roche Applied Science, Mannheim, Germany)) supplemented with phosphatase inhibitors (10 mM tetrasodium pyrophosphate, 17.5 mM *β-*glycerophosphate, and 100 mM NaF [25, 27]) for detection by antibodies directed against phospho-eIF2α (Cell Signaling Technology, Danvers, MA, USA), eIF2α (gift from Prof. Dr. D. Ron), KDEL (Enzo Life Sciences), GADD34 (ProteinTech, Chicago, IL, USA), puromycin (Millipore, Billerica, MA, USA), β-actin and GAPDH (CellSignalling), or in sample buffer (0.2 M Tris-HCl pH 6.8, 16% [v/v] glycerol, 4% [w/v] SDS, 4% [v/v] 2-mercaptoethanol, 0.003% [w/v] bromophenol blue) for detection by antibodies directed against (phospho-) p38 MAPK (both CellSignalling). The proteins in the samples were separated using a 10% SDS-PAGE gel and transferred onto a nitrocellulose membrane. After blocking with PBS containing 0.05% Tween-20 (v/v) and 5% skimmed-milk (w/v), the membrane was incubated overnight with the primary antibody (1:1000) in TBS with 0.05% Tween-20 (v/v) and 5% BSA (w/v) at 4°C. Next, the membrane was incubated with HRP-labelled anti-mouse or anti-rabbit antibody (Sigma) in blocking buffer for 1 hour and developed using ECL (ThermoScientific).

### Quantitative reverse-transcriptase polymerase chain reaction (qPCR)

Total RNA was isolated using Qiagen RNeasy mini kit (Qiagen/Westburg, Leusden, the Netherlands). Quantitative reverse-transcriptase polymerase chain reaction (qPCR) was performed as described previously [68] using the primer pairs as defined in Table 2. Relative mRNA concentrations of *RPL13A* and *ATP5B* (GeNorm, PrimerDesign Ltd., Southampton, UK) were used as housekeeping genes for human genes and *Actb* (β-actin) and *Sdha* for mouse genes to calculate normalized expression.

**Table 2:** qPCR primers.

Name	Forward primer	Reverse primer	Melting temp (°C)	Ref.
**HUMAN**				
CHOP	5’ GCACCTCCCAGAGCCCTCACTCTCC 3’	5’ GTCTACTCCAAGCCTTCCCCCTGCG 3’	62	[68]
GADD34	5’ ATGTATGGTGAGCGAGAGGC 3’	5’ GCAGTGTCCTTATCAGAAGGC 3’	62	[71]
GRP78	5’ CGAGGAGGAGGACAAGAAGG 3’	5’ CACCTTGAACGGCAAGAACT 3’	62	[72]
XBP1s	5’ TGCTGAGTCCGCAGCAGGTG 3’	5’ GCTGGCAGGCTCTGGGGAAG 3’	62	[68]
**MOUSE**				
Actb	5’ TCCTGGCCTCACTGTCCA 3’	5’ GTCCGCCTAGAAGCACTTGC 3’	59	[73]
Chop	5’ GGAGCTGGAAGCCTGGTATGA G 3’	5’ GCAGGGTCAAGAGTAGTGAAGG 3’	59	[56]
Gadd34	5’ CCCGAGATTCCTCTAAAAGC 3’	5’ CCAGACAGCAAGGAAATGG 3’	59	[74]
Sdha	5’ TTGCTACTGGGGGCTACGGGC 3’	5’ TGACCATGGCTGTGCCGTCC 3’	59	-
Xbp1s	5’ CTGAGTCCGAATCAGGTGCAG 3’	5’ GTCCATGGGAAGATGTTCTGG 3’	59	[75]

### ELISA

IL-8 was measured using commercially available ELISA kit according to manufacturer’s instructions (Sanquin, Amsterdam, the Netherlands).

### Cytotoxicity assays

LDH release was measured with a LDH-cytotoxicity colorimetric assay kit following manufacturer’s instructions (Biovision, Milpitas, CA, USA). Thiazolyl blue tetrazolium bromide (MTT; Sigma) was dissolved in a 5 mg/ml stock concentration in sterile water and cells were incubated with a 1:10 dilution for 2 hours at 37°C. Next, the water-insoluble formazan formed from MTT in viable cells was dissolved in isopropanol for 10 min before the absorbance was read at 570 nm wavelength.

### Electric cell-sensing impedance sensing

Epithelial barrier function was measured using ECIS (Applied Biophysics, Troy, NY, USA) as described previously [69]. Resistance was measured at 1000 Hz and cells were stimulated with CM-PAO1 when the resistance was stable.

### Statistical analysis

Results are expressed as mean ± SEM. Data were analysed using one- or two-way analysis of variance (ANOVA) and corrected with the Bonferroni *post-hoc* test. Differences with *P*-values <0.05 were considered to be statistically significant.

## Supporting Information

S1 Fig Conditioned medium of strain PAO1 causes disruption of the epithelial barrier function. A. Time- and dose-dependent decrease in epithelial resistance measured by ECIS. Primary bronchial epithelial cells were cultured on golden electrodes and epithelial resistance was measured every 5 minutes at 1000 Hz. Values are displayed as a relative number of the resistance at time point 0 (n = 3; mean ± SEM). B. Trypan blue staining of primary bronchial epithelial cells incubated for 0 or 12 hours with CM-PAO1.
(TIF)

S2 Fig Exotoxin A does not elicit an ER stress response in 16HBE cells. A. Normalised expression levels of *spliced XBP1*, *CHOP*, *GADD34* and *GRP78* mRNA in 16HBE cells after stimulation with 0, 1 or 10 ng/ml *P*. *aeruginosa* exotoxin A (ETA) (n = 3; mean ± SEM). All values are normalised to the housekeeping genes *RPL13A* and *ATP5B*. B. Phosphorylation of eIF2α (p-EIF2α) in 16HBE cells after stimulation with 0, 1 or 10 ng/ml *P*. *aeruginosa* exotoxin A (ETA). Total eIF2α serves as a loading control. C. Normalised expression levels of *spliced XBP1*, *CHOP*, *GADD34* and *GRP78* mRNA in 16HBE cells in 16HBE cells after stimulation with CM-PAO25, CM-PAN8 or CM-PAN8+Fe^3+^ (n = 3; mean ± SEM). All values are normalised to the housekeeping genes *RPL13A* and *ATP5B*. D. Western blot of a standard curve of purified AprA to semi-quantify the AprA content in undiluted CM-PAO25. * p<0.05, ** p<0.01, *** p<0.001 versus untreated (-) with a two-way repeated-measurements ANOVA (Bonferroni *post-hoc*).
(TIF)

S3 Fig Conditioned medium of strain PAO1 and PAO25 are comparable in inducing ER stress. A. Quantitation of pyocyanin in CM-PAO1 and CM-PAO25 (n = 3; mean ± SEM). B. Western blot for AprA levels present in CM-PAO1,-PAO25,-PAO25 cultured in the presence of iron (PAO25 + Fe^3+^),-PAN8 and-PAN11 (representative of n = 3). C. Splicing of *XBP1*, and *CHOP*, *GADD34* and *GRP78* mRNA levels in 16HBE cells treated with CM-PAO1 or CM-PAO25 (n = 3; mean ± SEM). * p<0.05, ** p<0.01, *** p<0.001 versus untreated (-) with a one-way repeated-measurements ANOVA (Bonferroni *post-hoc*).
(TIF)

S4 Fig CHOP can be regulated via the ER stress independent ISR. A. IL-8 release of 16HBE cells after treatment with CM-PAO1 in the presence of 30 μM 4μ8C, a selective inhibitor of the ER stress responsive kinase IRE1α (n = 3; mean ± SEM). B. *Chop* and *Gadd34* mRNA induction in *Chop* ^+/+^ or *Chop* ^*-/-*^ MEFs exposed to CM-PAO1 for 8, 16 or 24 hours or tunicamycin (Tm) for 6 hours as a positive control (n = 3; mean ± SEM). All values are normalised to the housekeeping genes *Actb* and *Sdha*. C. Splicing of *XBP1* mRNA in wild-type MEFs after treatment as in B. (n = 3; mean ± SEM). All values are normalised to the housekeeping genes *Actb* and *Sdha*. D-I. *Chop* mRNA normalised expression in *Perk* ^*-/-*^, *eIF2α* ^*AA*^, *Atf4* ^*-/-*^, *Pkr* ^*-/-*^, *Gcn2* ^*-/-*^ and *Hri* ^*-/-*^ mouse embryonic fibroblasts (MEFs) treated as in A. (n = 3; mean ± SEM). All values are normalised to the housekeeping genes *Actb* and *Sdha*. J. *CHOP* mRNA levels in HeLa cells upon exposure to CM-PAO1 after knock-down of GCN2 or HRI with siRNA (n = 3; mean ± SEM). All values are normalised to the housekeeping genes *RPL13A* and *ATP5B*. K. *Gadd34* mRNA levels in wild-type MEFs after repletion of the cell culture medium with iron (Fe^3+^) when treated with CM-PAO1. The first column (- Fe^3+^,—CM-PAO1) reflects medium control cells, without adding or depleting iron from the cell culture medium (n = 3; mean ± SEM). All values are normalised to the housekeeping genes *Actb* and *Sdha*. * p<0.05, ** p<0.01, *** p<0.001 versus untreated (-) with a two-way repeated-measurements ANOVA (Bonferroni *post-hoc*).
(TIF)

S5 Fig HRI and GCN2 knock down in epithelial cells. *HRI* and *GCN2* expression in HeLa cells after transfection with two different siRNA for each gene. (n = 3; mean ± SEM). All values are normalised to the housekeeping genes *RPL13A* and *ATP5B*. * p<0.05, ** p<0.01, *** p<0.001 versus untreated (-) with a two-way repeated-measurements ANOVA (Bonferroni *post-hoc*).
(TIF)

S6 Fig Expression of GFP-tagged GADD34 in HeLa Tet-ON cells. A. Response of HeLa cells incubated for 24 hours with a range of doxycycline concentrations (n = 3). B. Time-dependent induction of GFP-tagged GADD34 in HeLa cells treated with 0.5 μg/ml doxycycline (n = 3).
(TIF)

S7 Fig Schematic overview. Secreted virulence factors of *P*. *aeruginosa* induce both the UPR and the ISR. UPR induction is dependent on the TAK1-p38 MAPK pathway, whereas the induction of the ISR is mediated via iron deficiency. In human bronchial epithelial cells, the UPR causes XBP1 splicing, and the induction of GRP78 and CHOP (all in red). Iron deficiency, most likely in part caused by sequestration of iron by secreted siderophores, leads to activation of GADD34 via the ER stress independent kinase HRI (in blue). The common pathway is displayed in purple. In our model, it seems unlikely that CHOP influences GADD34. It is yet unknown whether cells distinguish between the phosphorylation of eIF2α by different kinases, and thereby influence specific induction of downstream targets.
(TIF)
