# Supplementary figures and images for: Virulence Factors of Pseudomonas aeruginosa Induce Both the Unfolded Protein and Integrated Stress Responses in Airway Epithelial Cells
Source: PLoS Pathog. 2015 Jun 17;11(6):e1004946. doi: 10.1371/journal.ppat.1004946 (PMC4471080; doi:10.1371/journal.ppat.1004946)

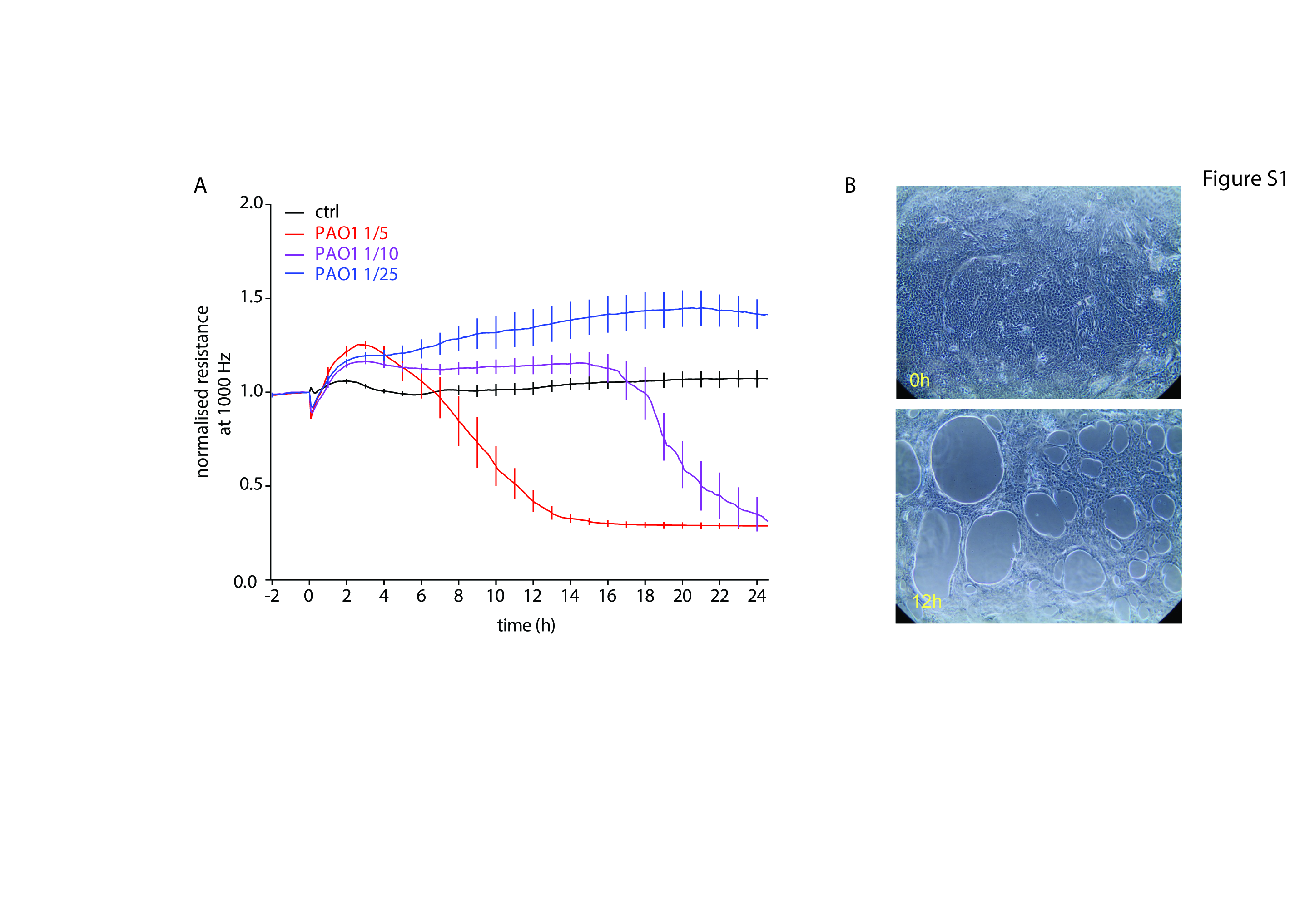

Supplement: S1 Fig — A. Time- and dose-dependent decrease in epithelial resistance measured by ECIS. Primary bronchial epithelial cells were cultured on golden electrodes and epithelial resistance was measured every 5 minutes at 1000 Hz. Values are displayed as a relative number of the resistance at time point 0 (n = 3; mean ± SEM). B. Trypan blue staining of primary bronchial epithelial cells incubated for 0 or 12 hours with CM-PAO1. (TIF) [file ppat.1004946.s001.tif]

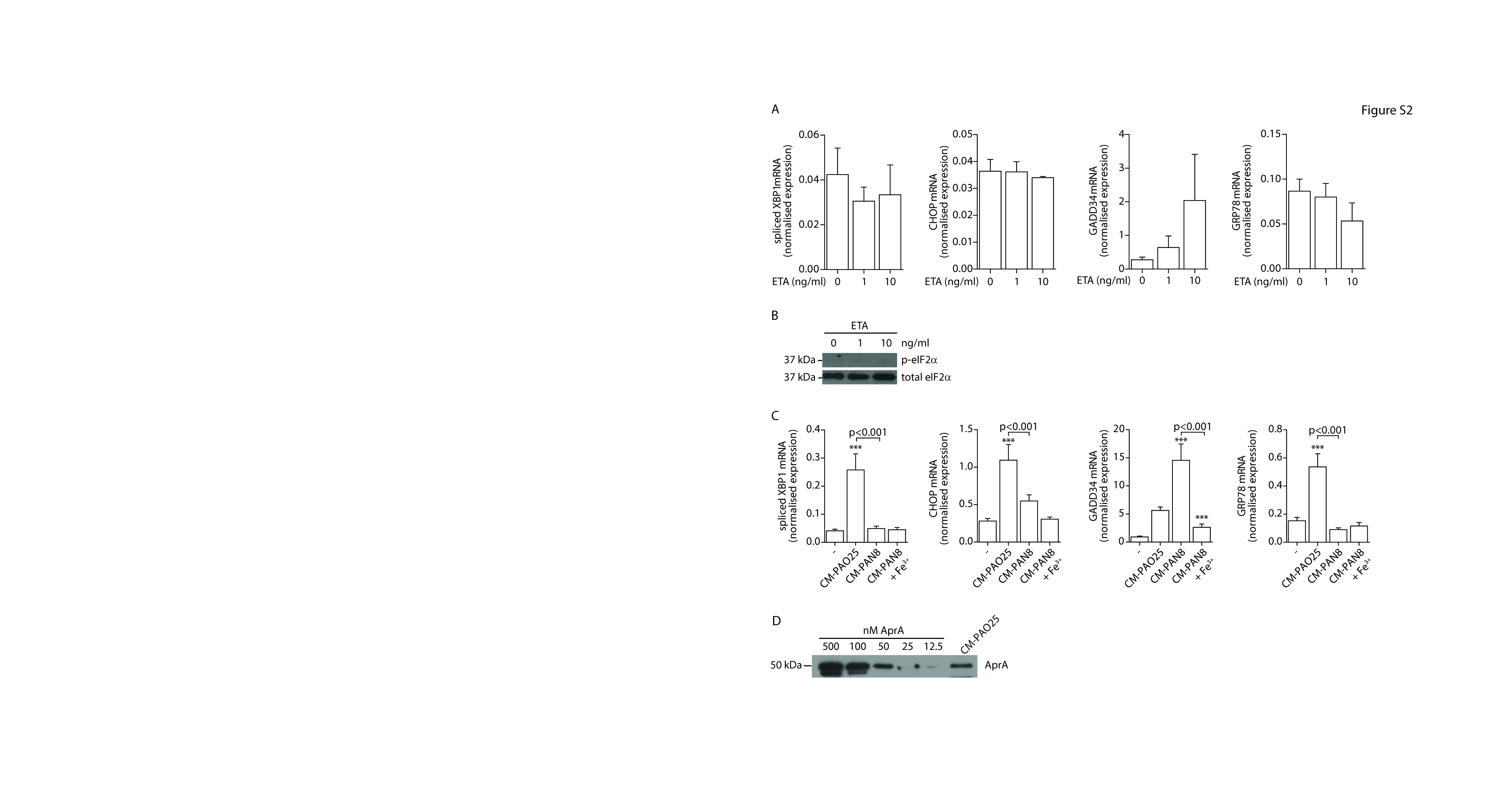

Supplement: S2 Fig — A. Normalised expression levels of spliced XBP1, CHOP, GADD34 and GRP78 mRNA in 16HBE cells after stimulation with 0, 1 or 10 ng/ml P. aeruginosa exotoxin A (ETA) (n = 3; mean ± SEM). All values are normalised to the housekeeping genes RPL13A and ATP5B. B. Phosphorylation of eIF2α (p-EIF2α) in 16HBE cells after stimulation with 0, 1 or 10 ng/ml P. aeruginosa exotoxin A (ETA). Total eIF2α serves as a loading control. C. Normalised expression levels of spliced XBP1, CHOP, GADD34 and GRP78 mRNA in 16HBE cells in 16HBE cells after stimulation with CM-PAO25, CM-PAN8 or CM-PAN8+Fe3+ (n = 3; mean ± SEM). All values are normalised to the housekeeping genes RPL13A and ATP5B. D. Western blot of a standard curve of purified AprA to semi-quantify the AprA content in undiluted CM-PAO25. * p<0.05, ** p<0.01, *** p<0.001 versus untreated (-) with a two-way repeated-measurements ANOVA (Bonferroni post-hoc). (TIF) [file ppat.1004946.s002.tif]

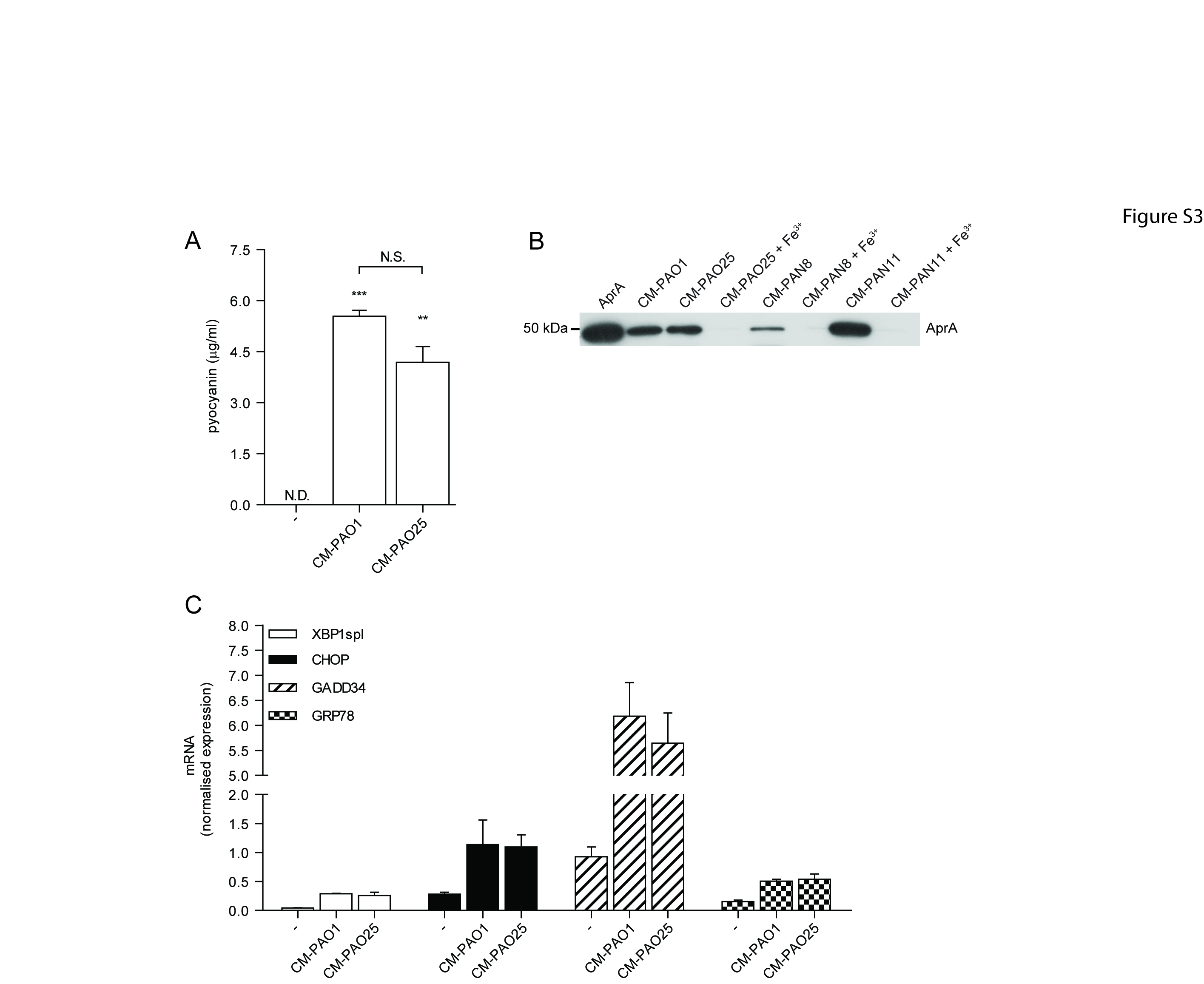

Supplement: S3 Fig — A. Quantitation of pyocyanin in CM-PAO1 and CM-PAO25 (n = 3; mean ± SEM). B. Western blot for AprA levels present in CM-PAO1,-PAO25,-PAO25 cultured in the presence of iron (PAO25 + Fe3+),-PAN8 and-PAN11 (representative of n = 3). C. Splicing of XBP1, and CHOP, GADD34 and GRP78 mRNA levels in 16HBE cells treated with CM-PAO1 or CM-PAO25 (n = 3; mean ± SEM). * p<0.05, ** p<0.01, *** p<0.001 versus untreated (-) with a one-way repeated-measurements ANOVA (Bonferroni post-hoc). (TIF) [file ppat.1004946.s003.tif]

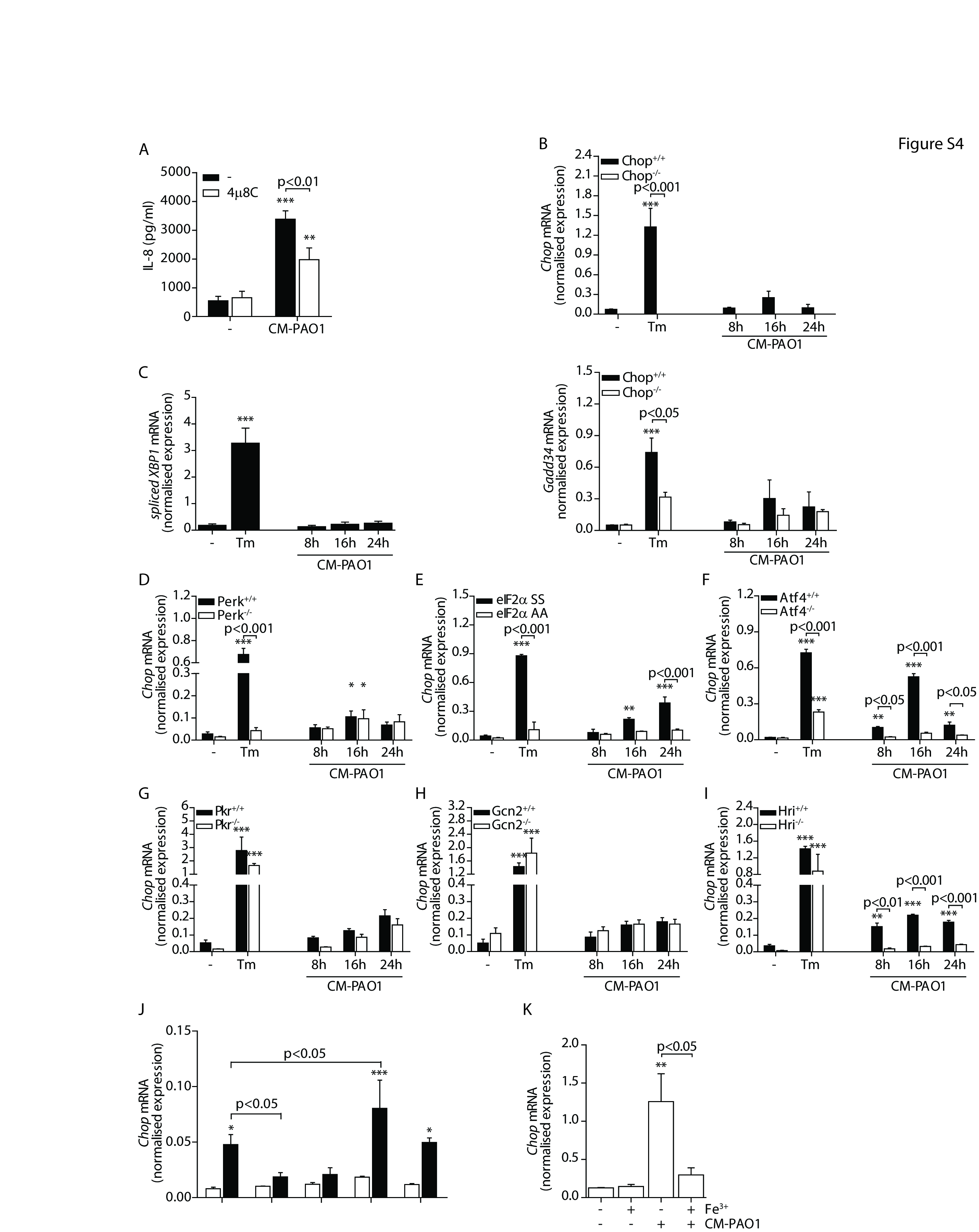

Supplement: S4 Fig — A. IL-8 release of 16HBE cells after treatment with CM-PAO1 in the presence of 30 μM 4μ8C, a selective inhibitor of the ER stress responsive kinase IRE1α (n = 3; mean ± SEM). B. Chop and Gadd34 mRNA induction in Chop +/+ or Chop -/- MEFs exposed to CM-PAO1 for 8, 16 or 24 hours or tunicamycin (Tm) for 6 hours as a positive control (n = 3; mean ± SEM). All values are normalised to the housekeeping genes Actb and Sdha. C. Splicing of XBP1 mRNA in wild-type MEFs after treatment as in B. (n = 3; mean ± SEM). All values are normalised to the housekeeping genes Actb and Sdha. D-I. Chop mRNA normalised expression in Perk -/-, eIF2α AA, Atf4 -/-, Pkr -/-, Gcn2 -/- and Hri -/- mouse embryonic fibroblasts (MEFs) treated as in A. (n = 3; mean ± SEM). All values are normalised to the housekeeping genes Actb and Sdha. J. CHOP mRNA levels in HeLa cells upon exposure to CM-PAO1 after knock-down of GCN2 or HRI with siRNA (n = 3; mean ± SEM). All values are normalised to the housekeeping genes RPL13A and ATP5B. K. Gadd34 mRNA levels in wild-type MEFs after repletion of the cell culture medium with iron (Fe3+) when treated with CM-PAO1. The first column (- Fe3+,—CM-PAO1) reflects medium control cells, without adding or depleting iron from the cell culture medium (n = 3; mean ± SEM). All values are normalised to the housekeeping genes Actb and Sdha. * p<0.05, ** p<0.01, *** p<0.001 versus untreated (-) with a two-way repeated-measurements ANOVA (Bonferroni post-hoc). (TIF) [file ppat.1004946.s004.tif]

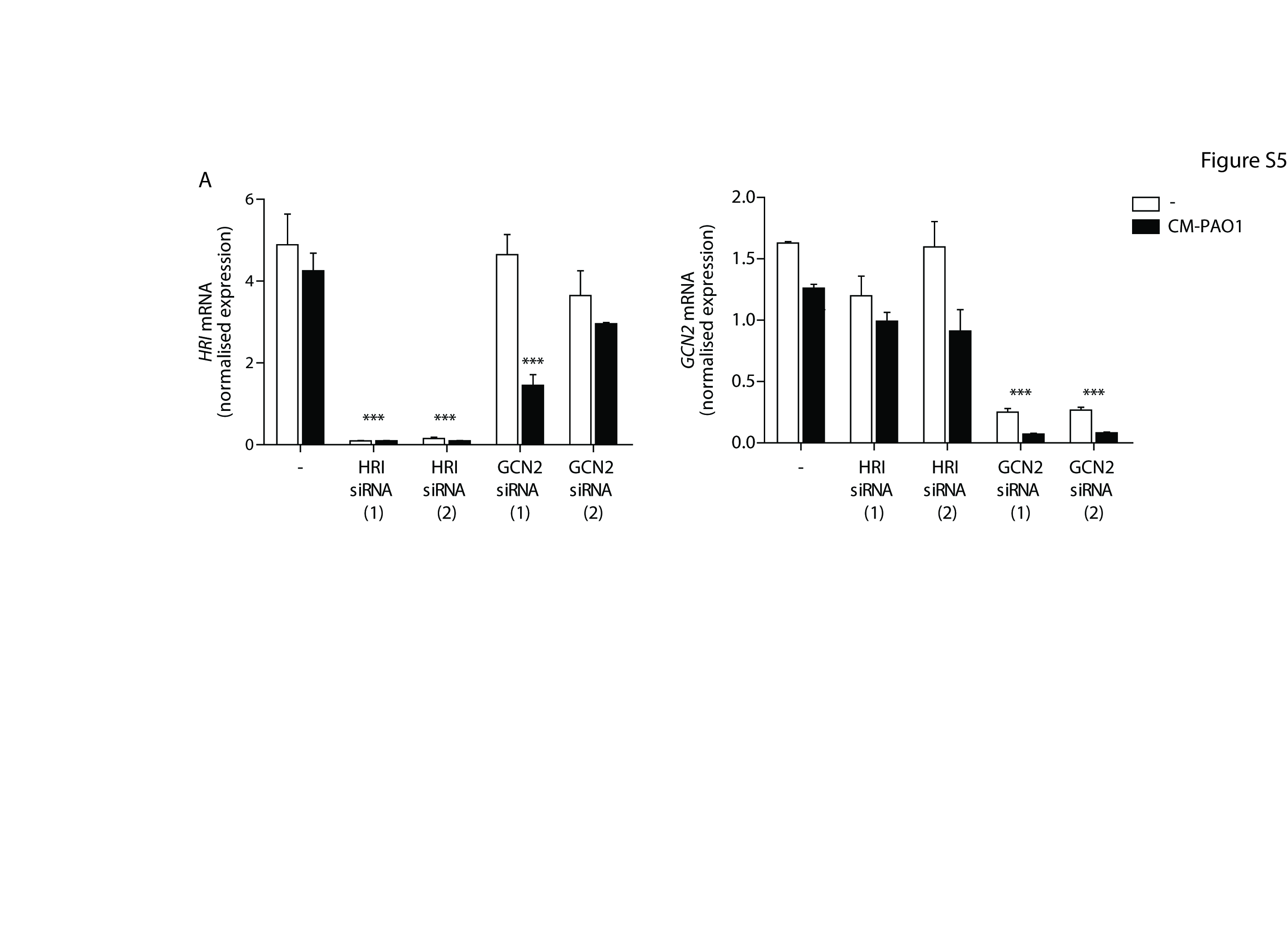

Supplement: S5 Fig — HRI and GCN2 expression in HeLa cells after transfection with two different siRNA for each gene. (n = 3; mean ± SEM). All values are normalised to the housekeeping genes RPL13A and ATP5B. * p<0.05, ** p<0.01, *** p<0.001 versus untreated (-) with a two-way repeated-measurements ANOVA (Bonferroni post-hoc). (TIF) [file ppat.1004946.s005.tif]

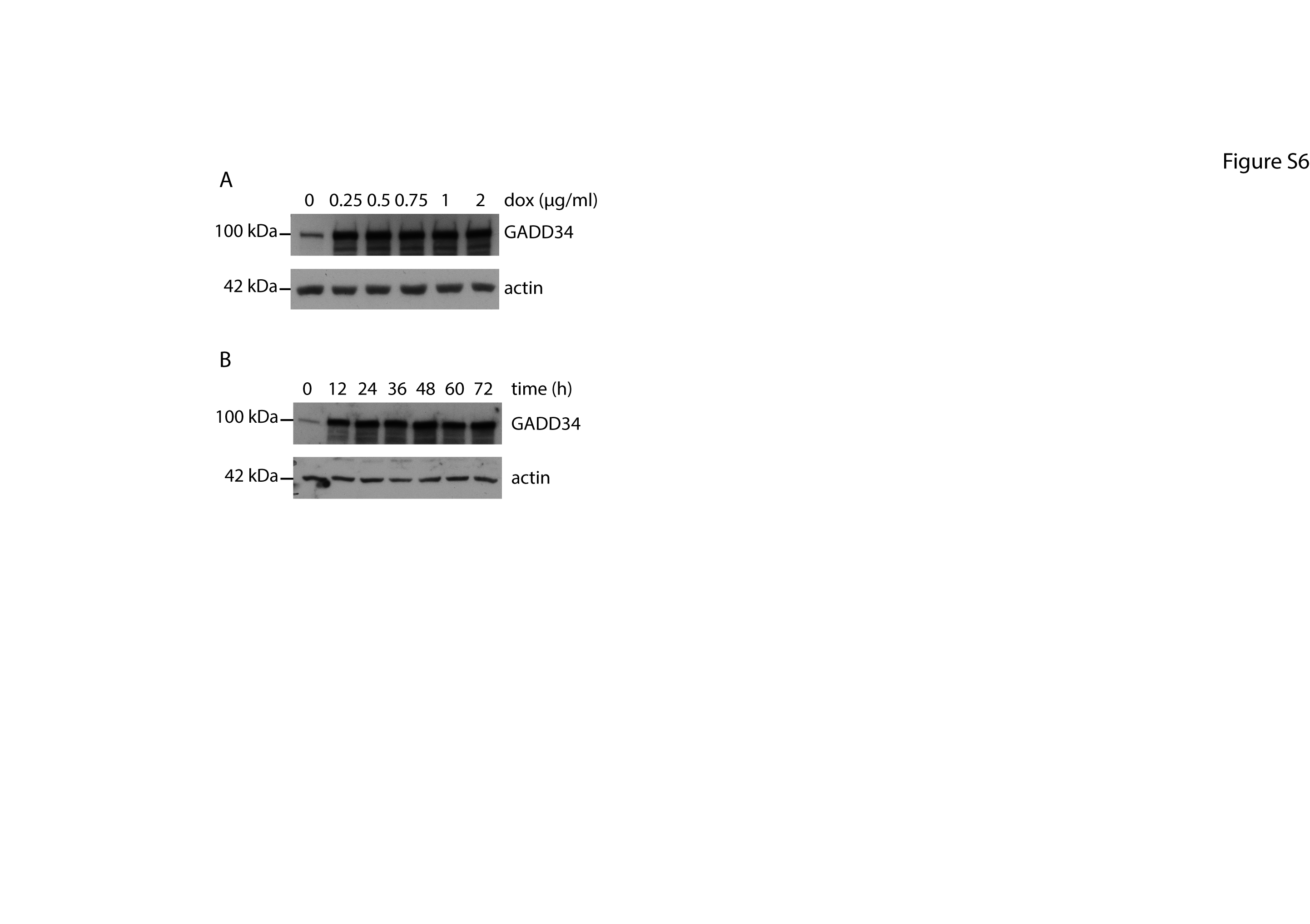

Supplement: S6 Fig — A. Response of HeLa cells incubated for 24 hours with a range of doxycycline concentrations (n = 3). B. Time-dependent induction of GFP-tagged GADD34 in HeLa cells treated with 0.5 μg/ml doxycycline (n = 3). (TIF) [file ppat.1004946.s006.tif]

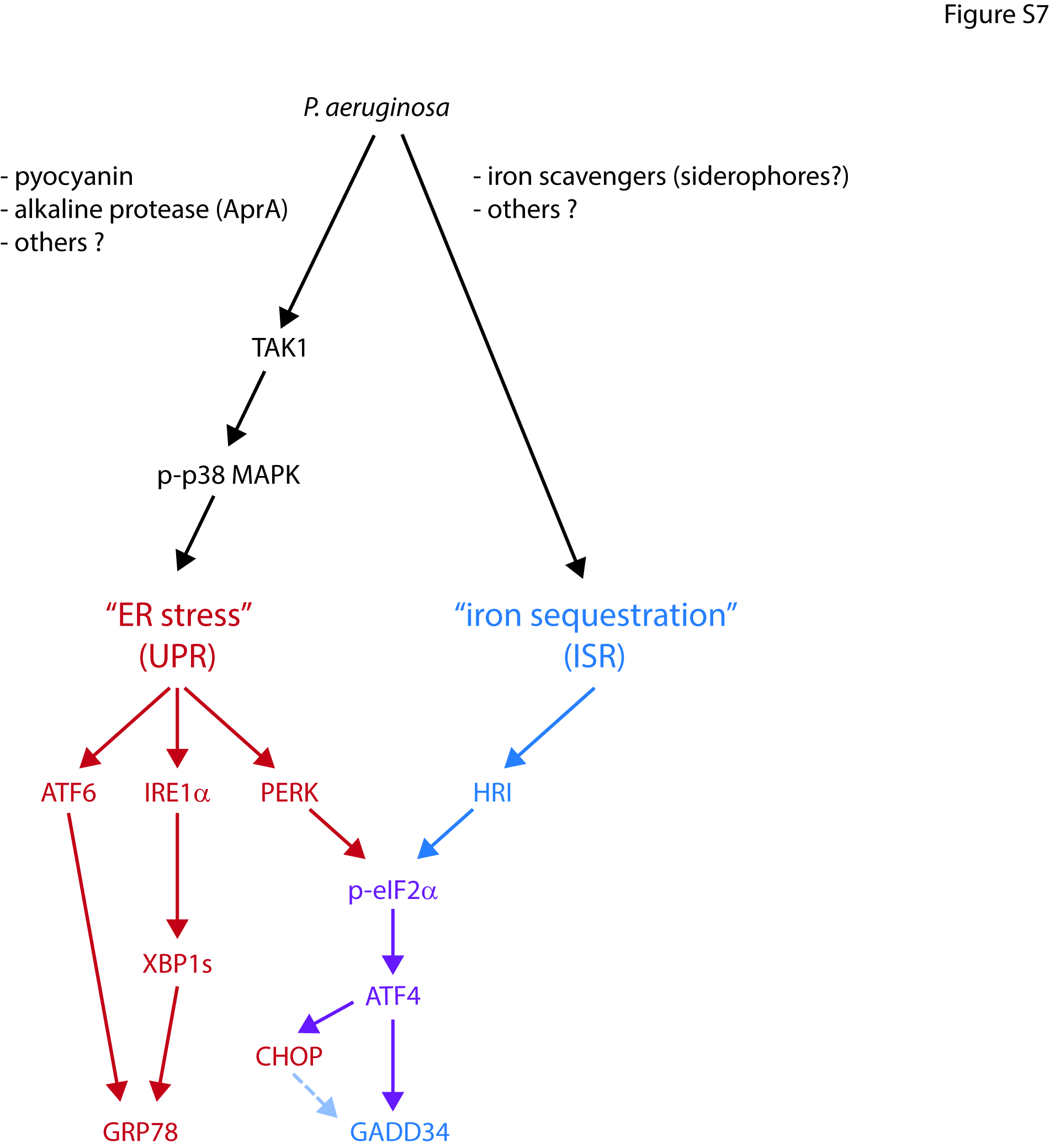

Supplement: S7 Fig — Secreted virulence factors of P. aeruginosa induce both the UPR and the ISR. UPR induction is dependent on the TAK1-p38 MAPK pathway, whereas the induction of the ISR is mediated via iron deficiency. In human bronchial epithelial cells, the UPR causes XBP1 splicing, and the induction of GRP78 and CHOP (all in red). Iron deficiency, most likely in part caused by sequestration of iron by secreted siderophores, leads to activation of GADD34 via the ER stress independent kinase HRI (in blue). The common pathway is displayed in purple. In our model, it seems unlikely that CHOP influences GADD34. It is yet unknown whether cells distinguish between the phosphorylation of eIF2α by different kinases, and thereby influence specific induction of downstream targets. (TIF) [file ppat.1004946.s007.tif]
